# Large-scale copy number analysis reveals variations in genes not previously associated with malignant pleural mesothelioma

**DOI:** 10.18632/oncotarget.22817

**Published:** 2017-12-01

**Authors:** Marieke Hylebos, Guy Van Camp, Geert Vandeweyer, Erik Fransen, Matthias Beyens, Robin Cornelissen, Arvid Suls, Patrick Pauwels, Jan P. van Meerbeeck, Ken Op de Beeck

**Affiliations:** ^1^ Center of Medical Genetics, University of Antwerp and Antwerp University Hospital, 2650 Antwerp, Belgium; ^2^ Center for Oncological Research, University of Antwerp, 2610 Antwerp, Belgium; ^3^ StatUa Center for Statistics, University of Antwerp, 2610 Antwerp, Belgium; ^4^ Department of Pulmonary Medicine, Erasmus Medical Center Cancer Institute, 3015 Rotterdam, The Netherlands; ^5^ Laboratory of Pathology, Antwerp University Hospital, 2650 Antwerp, Belgium; ^6^ Thoracic Oncology, Antwerp University Hospital, 2650 Antwerp, Belgium

**Keywords:** malignant pleural mesothelioma, copy number variations, low-pass whole genome sequencing, the cancer genome atlas, cancer census genes

## Abstract

Malignant pleural mesothelioma (MPM) is an aggressive tumor that is often causally associated with asbestos exposure. Comparative genomic hybridization techniques and arrays demonstrated a complex set of copy number variations (CNVs) in the MPM-genome. These techniques however have a limited resolution, throughput and flexibility compared to next-generation sequencing platforms.

In this study, the presence of CNVs in the MPM-genome was investigated using an MPM-cohort (*N* = 85) for which genomic microarray data are available through ‘The Cancer Genome Atlas’ (TCGA). To validate these results, the genomes of MPMs and matched normal samples (*N* = 21) were analyzed using low-pass whole genome sequencing on an ‘Illumina HiSeq’ platform. CNVs were detected using in-house developed analysis pipelines and frequencies of copy number loss and gain were calculated.

In both datasets, losses on chromosomes 1, 3, 4, 6, 9, 13 and 22 and gains on chromosomes 1, 5, 7 and 17 were found in at least 25% and 15% of MPMs, respectively. Besides the well-known MPM-associated genes, *CDKN2A, NF2* and *BAP1*, other interesting cancer-associated genes were listed as frequently involved in a copy number loss (e.g. *EP300, SETD2* and *PBRM1*). Moreover, four cancer-associated genes showed a high frequency of copy number gain in both datasets (i.e. *TERT*, *FCGR2B*, *CD79B* and *PRKAR1A*). A statistically significant association between overall survival and the presence of copy number loss in the *CDKN2A*-containing region was observed in the TCGA-set.

In conclusion, recurrent CNVs were detected in both datasets, occurring in regions harboring known MPM-associated genes and genes not previously linked to MPM.

## INTRODUCTION

Malignant pleural mesothelioma (MPM) is a rare and highly aggressive cancer originating from the mesothelial cells of the pleura [1]. A causal relationship between the development of MPM and exposure to asbestos has been demonstrated, with up to 80% of all patients being professionally exposed in the 30 to 40 years preceding the diagnosis [2]. Due to differences in historical asbestos import, consumption and ban, the incidence of MPM greatly varies between countries worldwide, ranging from seven patients per million inhabitants in Japan to 40 patients per million inhabitants in Australia [3]. Moreover, since asbestos is still being used in some non-Western and Western countries, MPM will remain a global health issue for decades to come [4]. Besides this increasing incidence, patients diagnosed with MPM still face a poor prognosis. The median overall survival time of untreated patients is six to ten months with a 5-year survival rate below 5%. Furthermore, current therapeutic options are limited and seem to provide only modest survival benefit [5, 6].

Genetic analyses have revealed genetic alterations in a number of genes in MPM. Of these, somatic inactivation of the tumor suppressor genes *CDKN2A*, *NF2* and *BAP1* seems to be the most prevalent [7–9]. Additionally, the presence of a complex and heterogeneous set of chromosomal copy number variations (CNVs) in MPM was described. Although no single MPM-specific alteration was observed, losses in chromosomes 1p, 4q, 9p, 13q, 14q and 22q were commonly noted using karyotype analyses and (microarray-based) comparative genomic hybridization techniques [10–19]. These techniques however have a limited resolution compared to highly sensitive next-generation sequencing platforms, which allow genome-wide detections in a high-throughput manner.

Here, we investigated the presence of CNVs in the MPM-genome using an MPM-cohort (*N* = 85), for which genomic microarray data are available through ‘The Cancer Genome Atlas’ (TCGA). These results were validated using low-pass whole genome sequencing (LP-WGS) on genomic DNA from paired tumor and normal samples of 21 MPM-patients. We found recurrent CNVs in several regions, harboring interesting cancer-associated genes.

## RESULTS

### The Cancer Genome Atlas

#### Copy number variations in MPM

**Figure 1:**
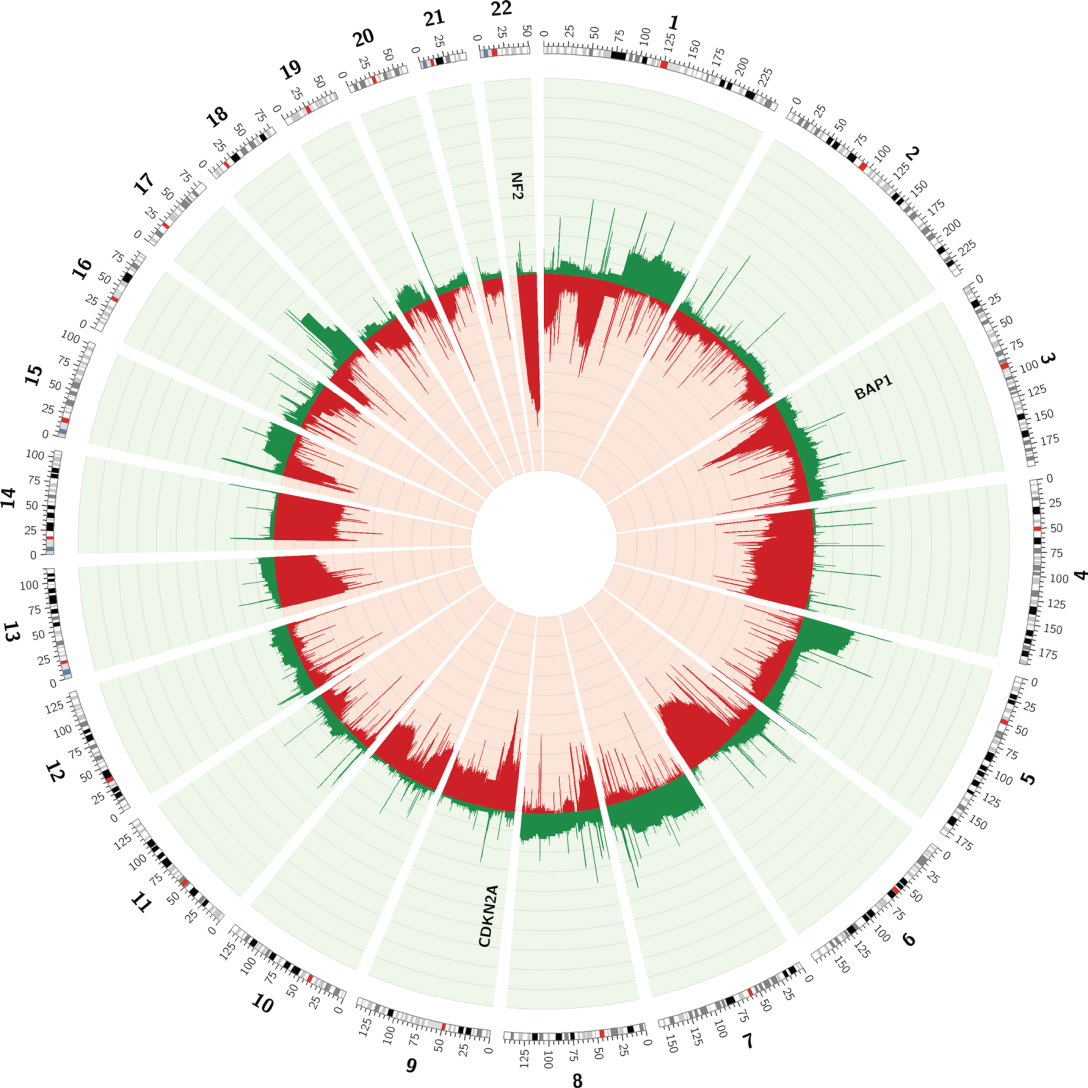
Circos plot of the CNVs observed in array data of 85 MPMs, available through TCGA Frequencies of copy number loss (red) and gain (green) are depicted for every chromosome position.

Segmented copy number data of 85 MPMs, available through the TCGA-website, were used to assess the MPM copy number profile (Table 1). In order to identify regions with recurrent CNVs in the 85 MPMs, frequencies of copy number loss and gain were calculated using the ‘Multi-intersect tool’ from ‘BEDtools’ (Figure 1) [20]. Large losses occurring in more than 25% of cases were identified on chromosomes 1 (p36.33-p36.13 and p31.1-p13.1), 3 (p22.2-p14.2), 4 (q13.1-q35.2), 6 (q14.1-q27), 9 (p22.2-p21.1), 13 (q11-q22.3), 14 (q11.1-q32.33) and 22 (q11.1-q13.33). Some regions on chromosome 22 were even lost in up to 75% of all studied MPMs. Gains occurred less frequently, with large regions on chromosomes 1 (q21.2-q44), 5 (p15.33-p11), 7 (p22.3-q11.21 and q11.21-q31.33) and 17 (q21.32-q25.1) exhibiting gains in more than 15% of MPMs.

**Table 1:** Clinical characteristics of the included MPM-patients (TCGA-set and LP-WGS-set)

Patient characteristics	Absolute amount (percentage)	
	TCGA (*N* = 85)	LP-WGS (*N* = 21)
Gender Male Female	69 (81%) 16 (19%)	19 (90%) 2 (10%)
Age at diagnosis Before or at the age of 60 After the age of 60 Unknown	32 (38%) 53 (62%) 0 (0%)	9 (43%) 10 (48%) 2 (10%)
History of asbestos exposure Yes No Unknown	54 (64%) 14 (16%) 17 (20%)	12 (57%) 0 (0%) 9 (43%)
Histologic diagnosis Epithelioid mesothelioma Non-epithelioid mesothelioma Unknown	56 (66%) 24 (28%) 5 (6%)	18 (86%) 3 (14%) 0 (0%)
Platinum/pemetrexed treatment prior to tissue collection No Yes	85 (100%) 0 (0%)	10 (48%) 11 (52%)
Time to death or last follow-up Less than 36 months More than 36 months Unknown	73 (86%) 11 (13%) 1 (1%)	16 (76%) 3 (14%) 2 (10%)
Vital status Dead Alive	56 (66%) 29 (34%)	15 (71%) 6 (29%)

In order to identify potentially interesting genes within regions exhibiting recurrent CNVs in the TCGA-dataset, the exact frequency of copy number loss and gain in the regions containing ‘Cancer census genes’ was listed [21]. The ‘top 20’ list of ‘Cancer census genes’ most frequently involved in a copy number loss contained some well-known MPM-associated genes (Table 2). Whereas *NF2* was lost in 62% of cases, *CDKN2A* was lost in 51% and *BAP1* in 44% of MPMs. However, the list also contained other genes, some even lost in a higher frequency of samples (e.g. *EP300*, *PDGFB*, *MKL1*, *MYH9*, *APOBEC3B* and *ZNF278*). *EP300* for example was located in a chromosomal region lost in 69% of all MPMs, being the highest reported frequency of copy number loss (Table 2). *EP300* encodes an histone acetyltransferase, regulating transcription via chromatin remodeling and influencing cell proliferation and differentiation [22, 23]. The frequency of copy number gain in regions containing ‘Cancer census genes’ was remarkably lower compared to the frequency of copy number loss (highest frequency of gain: 27% versus loss: 69%, Table 2). Nevertheless, regions containing some interesting ‘Cancer census genes’ on chromosomes 5, 1 and 17 showed copy number gain in a substantial number of patients. The region containing *TERT*, the gene encoding the catalytic component of the telomerase enzyme [24], exhibited a copy number gain in up to 27% of MPMs, being the most frequently reported copy number gain (Table 2).

**Table 2:** ‘Cancer census genes’ most frequently involved in a copy number loss or gain in the TCGA-data

‘Cancer census genes’ most frequently involved in a copy number loss^a^				‘Cancer census genes’ most frequently involved in a copy number gain^a^			
Gene name	Chromosome position	OG or TS^b^	Frequency loss (%)^c^	Gene name	Chromosome position	OG or TS^b^	Frequency gain (%)^c^
*EP300*	chr22:41,488,614-41,576,081	/	69.41	*TERT*	chr5:1,253,287-1,295,162	/	27.06
*PDGFB*	chr22:39,619,685-39,640,957	OG	68.24	*SDHA*	chr5:218,356-256,814	TS	24.71
*MKL1*	chr22:40,806,292-41,032,690	/	68.24	*DROSHA*	chr5:31,400,602-31,532,282	TS	23.53
*MYH9*	chr22:36,677,323-36,784,063	/	68.24	*IL7R*	chr5:35,856,977-35,879,705	/	22.35
*APOBEC3B*	chr22:39,378,404-39,388,784	OG/TS	64.71	*LIFR*	chr5:38,475,065-38,595,507	/	22.35
*ZNF278*	chr22:31,721,790-31,742,249	/	63.53	*FCGR2B*	chr1:161,632,905-161,648,444	/	21.18
*NF2*	chr22:29,999,545-30,094,589	TS	62.35	*CDC73*	chr1:193,091,088-193,223,942	TS	21.18
*MN1*	chr22:28,144,265-28,197,486	/	62.35	*PTPRC*	chr1:198,608,098-198,726,605	/	20.00
*CHEK2*	chr22:29,083,731-29,137,822	TS	62.35	*MDM4*	chr1:204,485,507-204,527,248	OG	20.00
*EWSR1*	chr22:29,663,998-29,696,515	/	62.35	*ELK4*	chr1:205,566,695-205,602,000	/	20.00
*BCR*	chr22:23,522,552-23,660,224	OG	60.00	*SLC45A3*	chr1:205,626,981-205,649,630	/	20.00
*SMARCB1*	chr22:24,129,150-24,176,705	/	60.00	*HLF*	chr17:53,342,321-53,402,426	OG	20.00
*MAPK1*	chr22:22,113,947-22,221,970	OG	57.65	*MSI2*	chr17:55,333,931-55,757,299	/	20.00
*CLTCL1*	chr22:19,167,712-19,279,239	TS	55.29	*RNF43*	chr17:56,431,038-56,494,931	/	20.00
*SEPT5*	chr22:19,704,743-19,711,102	/	55.29	*CLTC*	chr17:57,697,050-57,774,317	TS	20.00
*LZTR1*	chr22:21,336,558-21,353,326	TS	55.29	*PPM1D*	chr17:58,677,544-58,743,640	OG	20.00
*CDKN2A*	chr9:21,967,751-21,975,132	TS	50.59	*BRIP1*	chr17:59,756,547-59,940,920	TS	20.00
*SETD2*	chr3:47,057,898-47,205,467	TS	44.71	*CD79B*	chr17:62,006,098-62,009,704	OG	20.00
*BAP1*	chr3:52,435,020-52,444,121	TS	43.53	*DDX5*	chr17:62,494,374-62,502,484	OG	20.00
*NCKIPSD*	chr3:48,711,278-48,723,334	/	42.35	*AXIN2*	chr17:63,524,683-63,557,740	TS	20.00
*PBRM1*	chr3:52,579,368-52,713,739	TS	42.35	*PRKAR1A*	chr17:66,507,921-66,529,570	/	20.00

^a^The 20 ‘Cancer census genes’ most frequently involved in a copy number loss or gain were identified. However, as some ‘Cancer census genes’ showed exactly the same frequency of loss or gain, this list can contain more than 20 genes.

^b^Classified as an oncogene or tumor suppressor gene according to the ‘Cancer census gene’ list.

^c^When a ‘Cancer census gene’ was spread over multiple segments, the frequency of the segment containing the largest part of the gene was considered.

OG: oncogene; TS: tumor suppressor gene

#### Association with clinical and histological parameters

**Figure 2:**
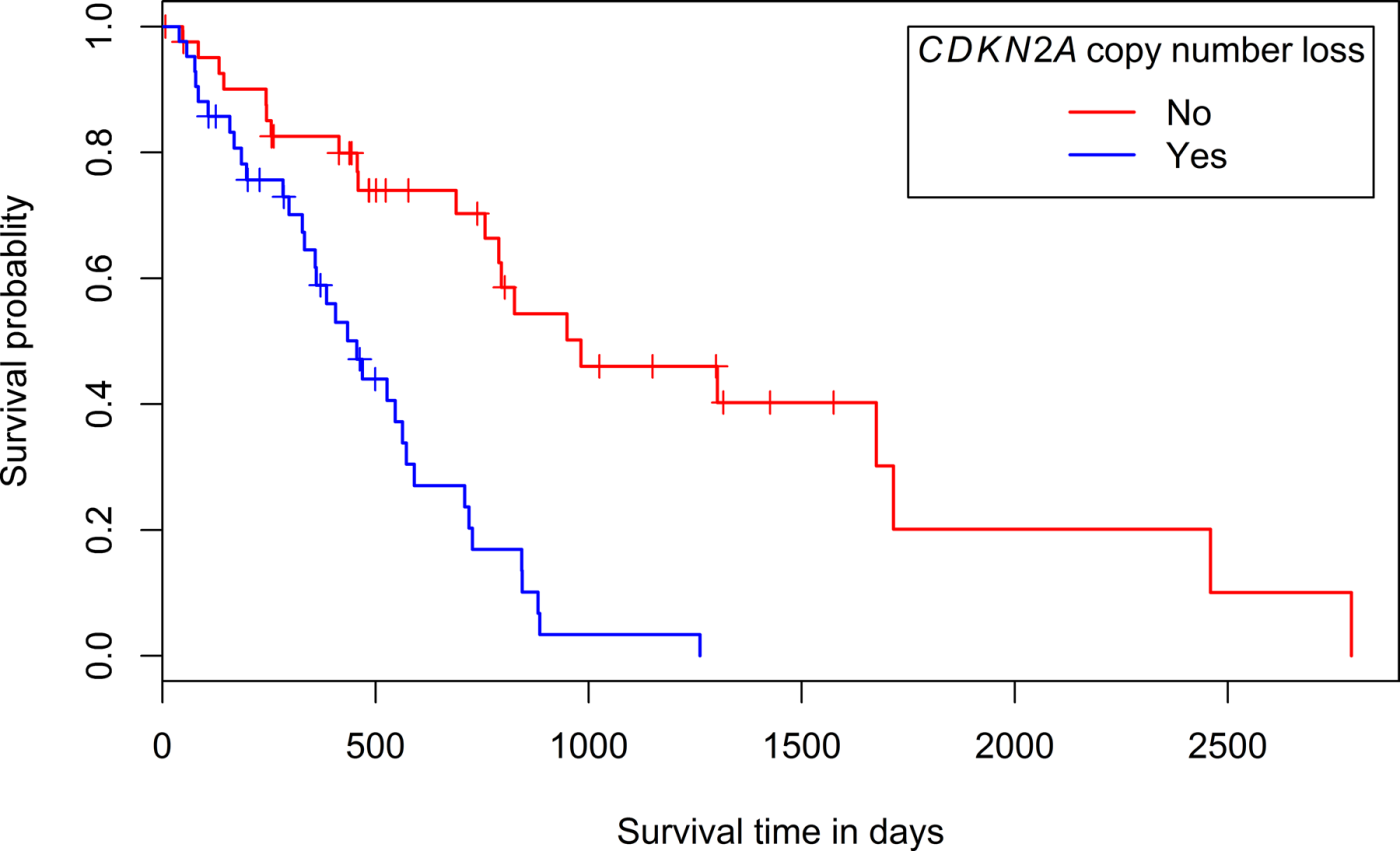
Kaplan-Meier plot of the overall survival according to the presence or absence of copy number loss in the chromosomal region containing *CDKN2A*

Associations between clinicopathological parameters and the presence of copy number loss (segment mean ≤ -0.25) or gain (segment mean ≥ 0.25) in the regions containing the most frequently involved ‘Cancer census genes’ (Table 2) were investigated. When for a certain sample a gene was spread over multiple segments with different segment means, this sample was not considered when examining potential associations. No statistically significant associations with gender, age at diagnosis (before or after the age of 60), asbestos exposure or histological diagnosis (epithelioid or non-epithelioid) were found (Supplementary Tables 1 and 2, depicting the *p*-values for the investigated associations). However, a statistically significant association was found between a survival less than 36 months and the presence of copy number loss in the segment containing *CDKN2A* (*p*-value: 0.01). Moreover, a univariate survival analysis showed a significantly longer survival time for patients with tumors without copy number loss in the segment containing *CDKN2A* (*p*-value: 4.54e^-6^, median survival of 982 days versus 456 days for patients with tumors with *CDKN2A* loss, Figure 2). Univariate analyses of the prognostic effect of gender, histologic subtype and age at diagnosis were not significant (*p*-values: 0.446; 0.0895 and 0.382 respectively). A non-significant trend towards an association between age at diagnosis (younger or older than 60 years) and the presence of copy number gain in the segment containing *TERT* was identified (*p*-value: 0.07).

### Low-pass whole genome sequencing

#### Copy number variations in MPM

To validate the results we obtained via the TCGA-dataset, 21 MPMs and matched normal samples were assessed for CNVs using LP-WGS (Table 1). For this analysis, the genome was divided in 50 kb-bins and for every bin, the log_2_-ratio comparing tumor versus normal sample was determined (Supplementary Figure 1, depicting the copy number profile of a representative sample pair). As in the data obtained using the TCGA-set, it was observed that copy number losses occurred more frequently in these MPMs compared to copy number gains.

**Figure 3:**
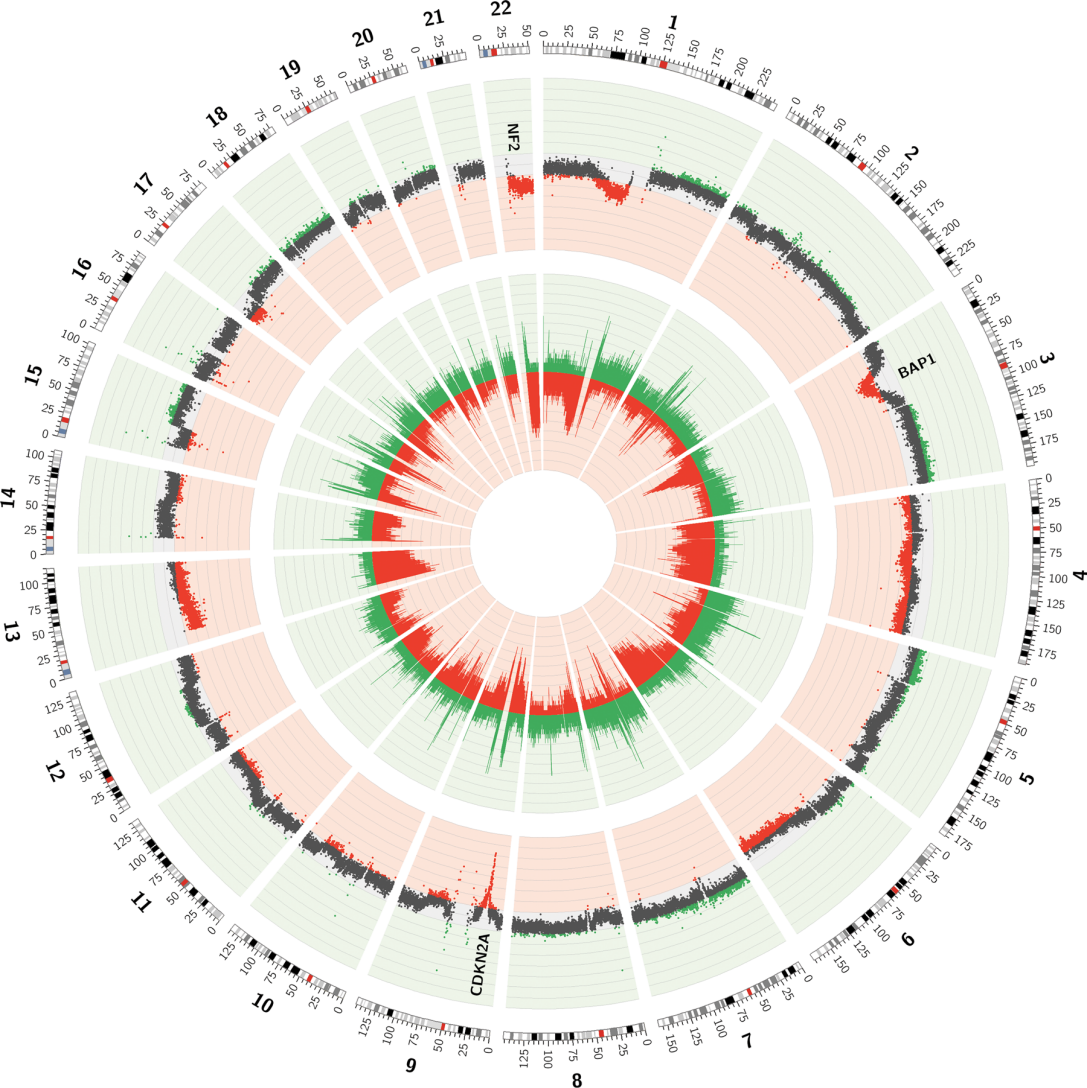
Circos plot of the CNVs observed in low-pass whole genome data of 21 MPMs The inner layer represents the frequency of copy number loss (red) and gain (green) in every 50 kb-bin. The outer layer represents the mean log_2_-ratio for every 50 kb-bin over the 21 tumor/normal sample pairs. Mean log_2_-ratios smaller than -0.10 or bigger than 0.10 are depicted in red or green respectively.

In order to identify regions with recurrent CNVs in the 21 MPMs, two different approaches were followed. A first strategy was based on calculating the frequencies of both copy number loss and gain in each of the 50 kb-bins. Doing so, regions with recurrent gains and losses were observed (Figure 3). Large losses occurring in more than 25% of cases were identified on parts of chromosomes 1 (p31.1-p11.2), 3 (p22.3-p14.1), 4 (p16.3-p11 and q12-q35.2), 6 (q15-q27), 9 (p23-p21.1), 13 (q11-q34), 17 (p13.3-p11.2) and 22 (q11.1-q13.33), with some regions being lost in up to 60% of all MPMs. Gains occurred less frequently, with regions less easy to demarcate on chromosomes 1 (q21.2-q44), 2 (p25.3-p22.3), 3 (q24-q29), 5 (p15.33-p11 and q11.1-q35.3), 7 (p22.3-p11.2 and q11.21-q36.3), 15 (q21.1-q26.3), 17 (q11.2-q25.3), 18 (p11.32-p11.21 and q11.1-q23) and 19 (p13.3-p12 and q11-q13.43) exhibiting gains in more than 15% of MPMs.

Similar as for the TCGA-data, the exact frequency of copy number gain and loss in the regions containing ‘Cancer census genes’ was assessed [21]. In contrast to the TCGA-data, the ‘top 20’ list of ‘Cancer census genes’ most frequently involved in a copy number loss in the LP-WGS-data did not contain *NF2*, *CDKN2A* or *BAP1* (Table 3). These genes were lost in 48%, 52% and 43% of MPMs respectively, which was not enough to rank them in the top. Six other ‘Cancer census genes’ however, were listed among the most frequently lost ones, both in the TCGA- and LP-WGS-dataset (i.e. *EP300*, *SETD2*, *PBRM1*, *CHEK2*, *MKL1* and *MAPK1*). As in the TCGA-data, *EP300* was the ‘Cancer census gene’ with the highest reported frequency of copy number loss, being in 71% of studied MPMs (Table 3). The frequency of copy number gain in regions containing ‘Cancer census genes’ was in line with that obtained in the TCGA-data and remarkably lower compared to the frequency of copy number loss (Table 3). Of the ‘Cancer census genes’ most frequently involved in a copy number gain, four were in common with those in the TCGA-data (i.e. *FCGR2B*, *TERT*, *CD79B* and *PRKAR1A*). *PMS2*, a gene encoding a component of the DNA mismatch repair system [25], was involved in a copy number gain in up to 33% of MPMs, being the most frequently reported copy number gain (Table 3).

**Table 3:** ‘Cancer census genes’ most frequently involved in a copy number loss or gain in the LP-WGS-data

‘Cancer census genes’ most frequently involved in a copy number loss^a^				‘Cancer census genes’ most frequently involved in a copy number gain^a^			
Gene name	Chromosome position	OG or TS^b^	Frequency loss (%)^c^	Gene name	Chromosome position	OG or TS^b^	Frequency gain (%)^c^
*EP300*	chr22:41,488,614-41,576,081	/	71.43	*PMS2*	chr7:6,012,870-6,048,737	/	33.33
*SETD2*	chr3:47,057,898-47,205,467	TS	66.67	*FCGR2B*	chr1:161,632,905-161,648,444	/	23.81
*PBRM1*	chr3:52,579,368-52,713,739	TS	66.67	*EIF4A2*	chr3:186,501,361-186,507,685	/	23.81
*ROS1*	chr6:117,609,530-117,747,018	OG	66.67	*TERT*	chr5:1,253,287-1,295,162	/	23.81
*ZNF198*	chr13:20,532,810-20,665,968	/	66.67	*HNRNPA2B1*	chr7:26,229,556-26,240,413	/	23.81
*RB1*	chr13:48,877,883-49,056,026	TS	66.67	*EGFR*	chr7:55,086,725-55,275,031	/	23.81
*CHEK2*	chr22:29,083,731-29,137,822	TS	66.67	*MET*	chr7:116,312,459-116,438,440	OG	23.81
*TRIM33*	chr1:114,935,399-115,053,781	/	61.90	*RAD21*	chr8:117,858,173-117,887,105	/	23.81
*CACNA1D*	chr3:53,529,076-53,846,492	OG	61,90	*KLF6*	chr10:3,818,188-3,827,473	/	23.81
*FLT3*	chr13:28,577,411-28,674,729	OG	61.90	*NAB2*	chr12:57,482,677-57,489,259	OG	23.81
*FOXO1*	chr13:41,129,801-41,240,734	OG/TS	61.90	*MLLT6*	chr17:36,861,873-36,886,056	/	23.81
*MKL1*	chr22:40,806,292-41,032,690	/	61.90	*CIC*	chr19:42,788,817-42,799,949	OG/TS	23.81
*EPS15*	chr1:51,819,935-51,984,995	/	57.14	*FAM131B*	chr7:143,050,493-143,059,840	/	19.05
*WHSC1*	chr4:1,873,123-1,983,934	/	57.14	*PLAG1*	chr8:57,073,468-57,123,859	OG	19.05
*PTPN13*	chr4:87,515,468-87,736,328	TS	57.14	*CHCHD7*	chr8:57,124,315-57,131,176	/	19.05
*RAP1GDS1*	chr4:99,182,527-99,365,012	/	57.14	*RECQL4*	chr8:145,736,667-145,743,210	/	19.05
*FBXW7*	chr4:153,242,410-153,456,185	/	57.14	*NUTM2B*	chr10:81,462,983-81,472,513	/	19.05
*FAT1*	chr4:187,508,937-187,644,987	TS	57.14	*NUTM2A*	chr10:88,985,205-88,994,733	/	19.05
*NFIB*	chr9:14,081,842-14,314,045	/	57.14	*ETNK1*	chr12:22,778,076-22,843,608	/	19.05
*MLLT3*	chr9:20,344,968-20,622,514	OG	57.14	*DICER1*	chr14:95,552,565-95,608,085	TS	19.05
*BRCA2*	chr13:32,889,617-32,973,809	TS	57.14	*CD79B*	chr17:62,006,098-62,009,704	OG	19.05
*LHFP*	chr13:39,917,029-40,177,356	/	57.14	*PRKAR1A*	chr17:66,507,921-66,529,570	/	19.05
*LCP1*	chr13:46,700,058-46,756,459	/	57.14	*ZNF521*	chr18:22,641,888-22,932,214	/	19.05
*MAPK1*	chr22:22,113,947-22,221,970	OG	57.14				

^a^The 20 ‘Cancer census genes’ most frequently involved in a copy number loss or gain were identified. However, as some ‘Cancer census genes’ showed exactly the same frequency of loss or gain, this list can contain more than 20 genes.

^b^Classified as an oncogene or tumor suppressor gene according to the ‘Cancer census gene’ list.

^c^For ‘Cancer census genes’ smaller than 50 kb, the frequency of copy number loss or gain in the 50 kb-region containing at least 90% of the gene was considered. ‘Cancer census genes’ smaller than 50 kb, that were not located for at least 90% in one bin, were excluded from this analysis. For the analysis of ‘Cancer census genes’ bigger than 50 kb, additional bins with the exact chromosomal location of these genes were analyzed.

OG: oncogene; TS: tumor suppressor gene

A second and novel strategy to determine regions with recurrent CNVs in the 21 MPMs was based on calculating the mean log_2_-ratio for every 50 kb-bin over the 21 sample pairs (Figure 3). Although this strategy precludes the identification of regions exhibiting both losses and gains in different tumors, these regions are less likely to be important in MPM-tumorigenesis. As a result, a sharper focus on the most interesting regions is obtained. In order to statistically summarize this information, a one-sample *t*-test was performed for every 50 kb-bin, with the null hypothesis assuming a mean log_2_-ratio of 0. In regions with mean log_2_-ratios different from 0, *p*-values were not uniformly distributed and some even shifted towards the significance threshold. A Kolmogorov-Smirnov (K-S) test was performed to confirm the non-uniform distribution of these *p*-values. Regions in which the *p*-value of the K-S test was smaller than 10^–15^ were listed in Table 4. The negative logarithm of the *p*-value of the K-S test was plotted against chromosome position (Supplementary Figure 2, depicting the mean copy number profile over the 21 sample pairs).

**Table 4:** Chromosomal regions showing copy number loss or gain in LP-WGS-dataset

Copy number loss		Copy number gain	
Chromosome	Chromosomal region	Chromosome	Chromosomal region
1	p31.1-p11.2	1	q25.3-q32.2
3	p22.3-p14.1		q32.3-q42.13
4	p16.3-p16.1		q42.13-q43
	p16.1-p15.1		q43-q44
	p15.1-p12	2	p15-p14
	q13.1-q25		p14-p13.3
	q26-q28.1		q22.2-q22.3
	q28.1-q28.3		q22.3-q23.3
	q31.1-q31.21		q24.1-q24.2
	q31.21-q35.1		q31.1
6	q14.3-q15		q31.3-q32.1
	q16.1-q21		q32.1-q32.3
	q21-q27	3	q12.1-q13.13
9	p22.2-p21.2		q13.13-q13.31
	q21.2-q21.31		q21.1-q21.2
	q21.31-q22.1		q21.3-q22.1
	q22.31-q22.32		q24-q26.1
10	q23.1		q26.1-q26.33
	q23.31-q23.33		q26.33-q28
	q24.2-q24.31	5	p15.33-p15.2
	q25.1		p15.2-q11.1
11	q21-q22.1	6	p25.3-p25.2
	q22.3-q23.1	7	p22.1-p13
	q23.1-q23.2		p13-p11.2
13	q11-q34		q11.23-q21.12
17	p13.3-p11.2		q21.13-q21.3
22	q11.1-q13.31		q31.1-q31.31
			q32.2-q33
		8	q12.1
			q21.13
			q21.3-q22.3
		12	q14.1-q15
			q15-q21.1
		15	q21.3-q22.31
			q22.31-q23
		18	p11.32-p11.31
			p11.31-p11.21
			q12.1
			q12.2-q12.3
			q12.3-q21.1
			q21.1-q21.2
			q21.2-q21.31
			q21.31-q21.32
			q21.33-q22.1
			q22.2-q23
		20	q13.12-q13.13
			q13.2-q13.32

#### Association with clinical and histological parameters

Associations between clinicopathological parameters and the presence of copy number loss (log_2_-ratio ≤ −0.25) or gain (log_2_-ratio ≥ 0.25) in the regions containing the most frequently involved ‘Cancer census genes’ (Table 3) were tested. No statistically significant associations with gender, age at diagnosis (before or after the age of 60), histological diagnosis (epithelioid or non-epithelioid), survival (more or less than 36 months) or chemotherapeutic treatment before sample collection were found (Supplementary Tables 3 and 4, depicting the *p*-values for the investigated associations).

## DISCUSSION

### Recurrent CNVs are detected in TCGA- and LP-WGS-data

In the past, karyotype analyses and (microarray-based) comparative genomic hybridization techniques have been employed to reveal the presence of a complex and heterogeneous set of chromosomal CNVs in MPM [10–19]. However, these techniques have a limited resolution compared to highly sensitive next-generation sequencing platforms. Therefore, we performed LP-WGS on genomic DNA from 21 paired tumor and normal samples to validate the results we obtained using array data from 85 MPMs, available through TCGA.

Both in the sample set from TCGA and from LP-WGS, chromosomal regions exhibiting frequent copy number loss or gain were identified (Supplementary Figure 3, comparing the CNVs in both sample sets). Losses of regions on chromosomes 1 (p31.1-p13.1), 3 (p22.2-p14.2), 4 (q13.1-q35.2), 6 (q15-q27), 9 (p22.2-p21.1), 13 (q11-q22.3) and 22 (q11.1-q13.33) were found in at least 25% of MPMs, both in the TCGA-set and LP-WGS-sample set. Regions with recurrent copy number gain occurred less frequently. Nevertheless, gains were detected on chromosomes 1 (q21.2-q44), 5 (p15.33-p11), 7 (p22.3-p11.2 and q11.21-q31.33) and 17 (q21.32-q25.1) in more than 15% of MPMs in both sample sets. Next to these similarities, some differences between both sample sets could be noted. The most striking of these differences was a loss of regions on chromosomes 1 (p36.33-p36.13) and 14 (q11.1-q32.33) which was not present in our LP-WGS-sample set. Moreover, some copy number gains identified in the LP-WGS-sample set on chromosomes 2 (p25.3-p22.3), 3 (q24-q29), 5 (q11.1-q35.3), 18 (p11.32-p11.21 and q11.1-q23) and 19 (p13.3-p12 and q11-q13.43) were not that frequent in the TCGA-data. These differences might be explained by the fact that the TCGA-set was significantly bigger than LP-WGS-set (i.e. 85 versus 21 MPMs). Furthermore, different techniques with different resolutions were used to identify CNVs (i.e. SNP-array and LP-WGS). Whereas the ‘Affymetrix Genome-Wide SNP Array 6.0’ allowed the analysis of more than 906,600 markers for single nucleotide polymorphisms and more than 946,000 markers for CNVs (inter-marker distance below 700 bases), LP-WGS achieved an average genome-wide coverage of 1.21x.

### ‘Cancer census genes’ are located in regions exhibiting recurrent CNVs

In order to identify potentially interesting genes within the regions exhibiting recurrent CNVs, the exact frequency of copy number loss and gain in the regions containing ‘Cancer census genes’ was determined, both for the TCGA- and LP-WGS-sample set [21]. The inactivation of the tumor suppressor genes *CDKN2A*, *NF2* and *BAP1* is well documented in MPM. Hence, it was no surprise that the regions in which these genes are located were frequently involved in a copy number loss in both sample sets. Whereas *CDKN2A* was lost in 51% of TCGA-samples and 52% of in-house samples, *NF2* exhibited loss in 62% and 48%, and *BAP1* in 44% and 43% of TCGA- and in-house samples respectively. Although these frequencies were sufficient to rank these genes in the ‘top 20’ list of ‘Cancer census genes’ most frequently involved in a copy number loss in the TCGA-sample set, this was not the case for our in-house sample set. However, given the recurrent deletion of *CDKN2A* in MPM (in more than 50% of cases in both datasets), its detection could be useful in a diagnostic and therapeutic setting. Regarding MPM-diagnosis, the use of fluorescence *in situ* hybridization to detect the homozygous deletion of *CDKN2A* proved helpful to distinguish between malignant mesothelial cells and benign reactive mesothelial cells both in pleural effusion and tissue samples [26, 27]. Regarding MPM-therapy, inactivation of *CDKN2A* results in deregulation of CDK4 and CDK6, which makes MPMs good candidate responders to CDK4- and CDK6-inhibitory drugs. In hormone receptor-positive metastatic breast cancer, palbociclib, an inhibitor of CDK4 and CDK6, significantly improves progression-free survival [28]. Currently, the option of starting a phase II study with small molecule CDK-inhibitors in patients with refractory MPM is being investigated (NCT02187783). It should however be noted that also *RB1* was frequently involved in a copy number loss in the LP-WGS-sample set. As loss of RB1-function is reported to be a mechanism of resistance to CDK-inhibitors, a subset of MPMs might have to be excluded from trials aiming at proving the efficacy of CDK-inhibitors in MPM [29].

Both in the TCGA- and LP-WGS-sample set, other interesting cancer-associated genes were listed as being frequently involved in a copy number loss (Tables 2 and 3). Strikingly, both sample sets shared six genes in their ‘top 20’ list of most frequently lost ‘Cancer census genes’ (i.e. *EP300*, *SETD2*, *PBRM1*, *CHEK2*, *MKL1* and *MAPK1*). *EP300*, in both sample sets the ‘Cancer census gene’ with the highest reported frequency of copy number loss, encodes an histone acetyltransferase, important in cell proliferation and differentiation [22, 23]. EP300 has been reported to play a role in tumorigenesis, and inactivating mutations in *EP300* have been described in several solid tumor types (e.g. colorectal and gastric tumors) [30]. However, not much is known about the role of *EP300* in MPM. *SETD2*, encoding a member of the SET-domain family containing histone methyltransferases [31], and *PBRM1*, encoding a subunit of ATP-dependent chromatin remodeling complexes [32], have been recently linked to MPM. Not only mutations, gene fusions and splice alterations were described, also frequent minute deletions were found in these genes [19, 33, 34]. Moreover, silencing of *SETD2* or *PBRM1* was found to increase proliferation in a mesothelioma cell line [34]. Regarding the cancer-associated genes *CHEK2*, encoding a cell cycle checkpoint regulator; *MKL1*, encoding a protein amongst others involved in transducing signals from the cytoskeleton to the nucleus; and *MAPK1*, encoding an essential component of the MAP kinase signal transduction pathway, not much is known about their role in MPM. Yet, one study did report that a substantial amount of miRNAs, downregulated in MPM, targeted MAPK1, which might suggest that this molecule is overexpressed in MPM, in contrast to our results [35].

Although in both sample sets the frequency of copy number gain in regions containing ‘Cancer census genes’ was remarkably lower compared to the frequency of copy number loss, some interesting genes were among the most frequently gained ones (Tables 2 and 3). Moreover, both sample sets shared four genes in their ‘top 20’ list of ‘Cancer census genes’ most frequently involved in a copy number gain (i.e. *TERT*, *FCGR2B*, *CD79B* and *PRKAR1A*). *TERT*, the ‘Cancer census gene’ exhibiting the most frequent copy number gain in the TCGA-set, encodes the catalytic component of the telomerase enzyme. Telomerase expression is normally repressed in postnatal somatic cells resulting in progressive shortening of the telomeres. However, deregulation of telomerase expression in somatic cells can contribute to a replicative immortality, which is one of the ‘Hallmarks of Cancer’ [36]. In line with this function, TERT expression was detected in 99% of MPMs using immunohistochemistry and *in situ* hybridization [37]. Furthermore, TERT mRNA was found to be upregulated in MPM. Nonetheless, this upregulation was reported to be the result of mutations in the *TERT* promoter and not of gene copy number amplification [38]. Regarding the cancer-associated genes *FCGR2B,* encoding a low affinity receptor for the Fc-region of immunoglobulin gamma complexes; *CD79B*, encoding the immunoglobulin beta protein which is necessary for functioning of the B-cell antigen receptor; and *PRKAR1A*, encoding one of the regulatory subunits of the cAMP-dependent protein kinase, not much is known about their role in MPM. Strikingly, *PMS2*, the ‘Cancer census gene’ most frequently involved in a copy number gain in the LP-WGS-set, was not ranked among the most frequently gained genes in the TCGA-set. As this gene encodes a component of the DNA mismatch repair system [25], one would not expect a copy number gain of the region containing this gene. However, overexpression of PMS2 was previously reported to confer genetic instability and DNA-damage tolerance in prostate cancer [39, 40].

Regarding these results, it should be noted that the ‘Cancer census genes’ that are most frequently involved in a copy number loss or gain, are clustered in certain regions (Tables 2 and 3). For example, a substantial amount of the most lost genes in the TCGA-dataset are located on chromosome 22. As chromosome 22 is almost entirely lost in more than 60% of MPMs in this dataset, this is no surprise. Obviously, not all listed genes will be equally important in MPM-tumorigenesis, and some genes will only be listed as they are in the proximity of more important ones. This might explain why even some oncogenes (e.g. *MAPK1*) pop up. Only further functional studies can elucidate the role of each of the listed genes in the pathogenesis of MPM.

### *CDKN2A* loss is associated with a shorter overall survival

In the LP-WGS-set, no statistically significant associations between any of the investigated clinicopathological parameters and the presence of copy number loss or gain in regions with selected ‘Cancer census genes’ were found (Supplementary Tables 3 and 4, depicting the *p*-values for the investigated associations). In the TCGA-set however, a statistically significant association was found between an overall survival shorter than 36 months and the presence of copy number loss in the chromosomal segment containing *CDKN2A*, which was confirmed by a univariate survival analysis (Supplementary Table 1 and Figure 2). Differences in prognosis according to *CDKN2A* deletion status and CDKN2A (p16ink4a/p14ARF) protein expression were previously reported [41–45]. In several reports, a statistically significant survival advantage was found for patients with tumors without *CDKN2A* homozygous deletion [41, 42, 45]. Moreover, in studies by Dacic et al. and Kobayashi et al., loss of CDKN2A (p16ink4a) protein expression, as detected by immunohistochemistry, was shown to be associated with a poor prognosis. Whereas Dacic et al. also reported significant differences in survival according to the homozygous deletion status of *CDKN2A*, this was not mirrored by Kobayashi et al. [42, 43]. In a study by Walter et al., a survival difference was seen between patients with a low CDKN2A (p14ARF) mRNA-expression and patients with a high expression. Nevertheless, the association between overall survival and CDKN2A (p14ARF) mRNA-expression did not reach statistical significance [44]. Given the limited therapeutic options for MPM-patients, their modest benefit and sometimes substantial toxicity, identifying patients with a particularly poor prognosis can be beneficial. Hence, the potential utility of *CDKN2A* deletion in a prognostic setting holds promise for the future.

## MATERIALS AND METHODS

### TCGA-data collection

TCGA is a joint effort of the National Cancer Institute (NCI) and the National Human Genome Research Institute (NHGRI), that has generated comprehensive maps of the key genomic changes in 33 types of cancer (http://cancergenome.nih.gov/). Regarding MPM, TCGA holds data of 87 patients, including segmented copy number data (TCGA level 3 data, hg19.seg-files). For the latter, the original data files were generated using the ‘Affymetrix Genome-Wide SNP Array 6.0’ (Thermo Fisher Scientific, Waltham, MA, USA), and the files were analyzed using the ‘CopyNumberInferencePipeline’ in ‘GenePattern’ [46]. In each of the resulting files, segment means were normalized against a panel of several thousands of blood normal samples. Patient characteristics of the TCGA-patients are summarized in Table 1. Two patients in the TCGA MPM-cohort received neo-adjuvant treatment, whereas all others did not. Therefore, we chose to exclude these two patients from all analyses.

### Copy number profiling of TCGA-data

In order to identify recurrent copy number differences in the MPM-samples of which segmented copy number data were available through TCGA, frequencies of copy number loss and gain were calculated. In this respect, regions with segment means smaller or equal than -0.25 were considered as losses and regions with segment means bigger or equal than 0.25 were considered as gains. Using the ‘Multi-intersect tool’ from ‘BEDtools’ [20], chromosomal regions with recurrent copy number loss or gain in the 85 MPMs were identified, after which frequencies were calculated. In order to identify potentially interesting genes within regions exhibiting recurrent CNVs, the frequency of copy number loss and gain specifically in the regions containing ‘Cancer census genes’ was determined. The ‘Cancer census genes’ are genes with substantial published evidence in oncology. This list, containing 609 genes at the time of first analysis (accessed in November 2016), is regularly updated by the COSMIC team and can be found on their website [21].

### Patient samples collection and preparation

In order to validate the results obtained using TCGA-data, LP-WGS was performed on an independent MPM-cohort. This study was conducted with the approval of the ethical committee of the Antwerp University Hospital and the University of Antwerp (Reference numbers 14/8/73 & 16/23/248). Twenty-one MPM- and matched normal samples were obtained from the tumor bank of the Antwerp University Hospital (Biobank@UZA, Antwerp, Belgium; ID: BE71030031000, Belgian Virtual Tumorbank funded by the National Cancer Plan) and from the tissue bank of the Erasmus University Medical Center Rotterdam. Patient characteristics are summarized in Table 1. Non-tumor material consisted of cryopreserved blood lymphocytes, collected before or after surgery. When matched blood samples were not available, healthy lung or pleura tissue, removed during resection, was used. All tissue samples were collected in the operating room, immediately snap-frozen in liquid nitrogen and stored at –80°C. Diagnosis and tumor content were confirmed by histological examination of hematoxylin-eosin-stained 5 µm-sections. Histology of the tumor samples included epithelioid (*N* = 18), biphasic (*N* = 2) and epithelioid/desmoplastic (*N* = 1). DNA was extracted from each of the blood samples and from fifteen 10 µm-sections per tissue sample using the ‘QIAamp DNA Mini Kit’ (Qiagen, Hilden, Germany, Cat. No. 51304), according to the manufacturer’s instructions.

### Copy number profiling of LP-WGS-data

Genomic DNA was fragmented using a Covaris instrument (Covaris, Woburn, MA, USA) and sequencing libraries were generated using the ‘KAPA Library Preparation Kit’ (Roche, Basel, Switzerland, Cat. No. KK8230). Next, sample libraries were sequenced on an ‘Illumina HiSeq 1500 platform’ (Illumina, San Diego, CA, USA) in high output mode, generating 2 × 100 bp paired-end reads. This resulted in an average coverage of 1.21x, which enabled the detection of structural variants.

Sequencing reads were adapter trimmed and mapped to the UCSC human genome (GRCh37/hg19). The presence of CNVs in the samples was analyzed using in-house developed analysis pipelines. The algorithm divides the genome into non-overlapping 50 kb-bins and counts all mapped sequencing reads for each tumor and normal sample within each bin. After correction of read counts for local GC-content using lowess normalization, log_2_-ratios were calculated for every tumor and normal sample pair.

In order to identify recurrent copy number differences between tumor and normal samples, frequencies of copy number loss and gain were calculated for each of the 50 kb-bins. We used a log_2_-ratio threshold of -0.25 for chromosomal losses and 0.25 for copy number gains. Similar as for the TCGA-data, the frequency of copy number loss and gain specifically in the regions containing ‘Cancer census genes’ was assessed [21]. For ‘Cancer census genes’ smaller than 50 kb, the frequency of copy number loss and gain in the 50 kb-region containing at least 90% of the gene was considered. ‘Cancer census genes’ smaller than 50 kb, that were not located for at least 90% in one bin, were excluded from this analysis. To enable the analysis of ‘Cancer census genes’ bigger than 50 kb, additional bins with the exact chromosomal location of these genes were analyzed. Next to this frequency-based approach, the mean log_2_-ratio for each 50 kb-bin over the 21 sample pairs was determined. This is a novel approach enabling the identification of recurrent copy number differences between tumor and normal samples. Within each of the 50 kb-bins, a one-sample *t*-test was carried out, testing the null hypothesis that the mean log_2_-ratio within this bin equals 0. If all null hypotheses are true across all bins tested, it is expected that the *p*-values of these tests follow a uniform distribution with boundaries 0 and 1. This latter hypothesis was tested using the K-S test. One K-S test was carried out per sliding window of 50 *p*-values (coming from the one-sample *t*-test). The step width between the sliding windows was set to 25.

### Statistical analysis

To identify associations between clinicopathological parameters on the one hand and the presence of copy number loss or gain in regions containing selected genes on the other hand, a Pearson’s Chi-squared test with Yates’ continuity correction was performed. In case more than 20% of the cells had an expected count below five, a Fisher’s Exact test was used. Segment means (TCGA-data) and log_2_-ratios (LP-WGS-data) smaller or equal than -0.25 were considered as losses and values bigger or equal than 0.25 were considered as gains.

Parameters such as the presence of copy number loss or gain in regions containing selected genes, gender and histological subtype, were tested for association with overall survival using the log-rank test. In this respect, overall survival was defined as the time from initial pathologic diagnosis to the date of death or last follow-up. Survival curves were plotted using the method of Kaplan and Meier.

All p-values were based on a two-sided hypothesis, with p-values smaller or equal than 0.05 considered statistically significant. Decimal values were rounded to the nearest digit. Statistical analyses were carried out using the statistical software ‘R’ version 2.3.1. [47].

## CONCLUSIONS

Recurrent copy number losses and gains were identified in the TCGA-set and confirmed in an in-house sample set using LP-WGS. These CNVs occurred in regions harboring cancer-associated genes that are potentially useful in a diagnostic, therapeutic and prognostic setting.

## SUPPLEMENTARY MATERIALS FIGURES AND TABLES



## Abbreviations

CNV: Copy number variation
K-S: Kolmogorov-Smirnov
LP-WGS: Low-pass whole genome sequencing
MPM: Malignant pleural mesothelioma
TCGA: The Cancer Genome Atlas
