# Supplementary material for: Large-scale copy number analysis reveals variations in genes not previously associated with malignant pleural mesothelioma
Source: Oncotarget. 2017 Dec 1;8(69):113673–86. doi: 10.18632/oncotarget.22817 (PMC5768355; doi:10.18632/oncotarget.22817)
Supplement: Supplementary file 1 [file oncotarget-08-113673-s001.pdf]

Large-scale copy number analysis reveals variations in genes not previously associated with malignant pleural mesothelioma

SUPPLEMENTARY MATERIALS

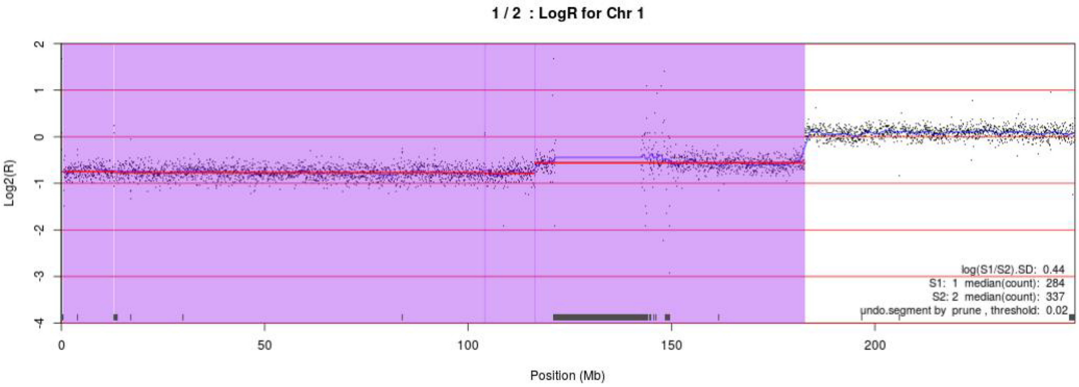

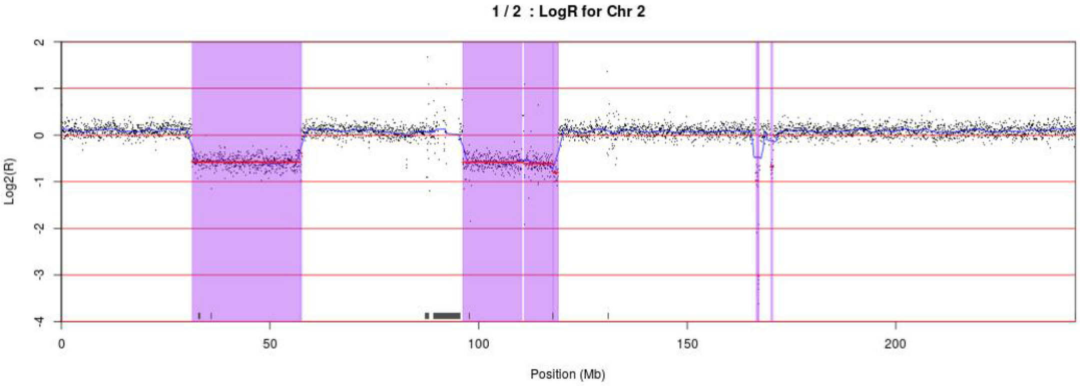

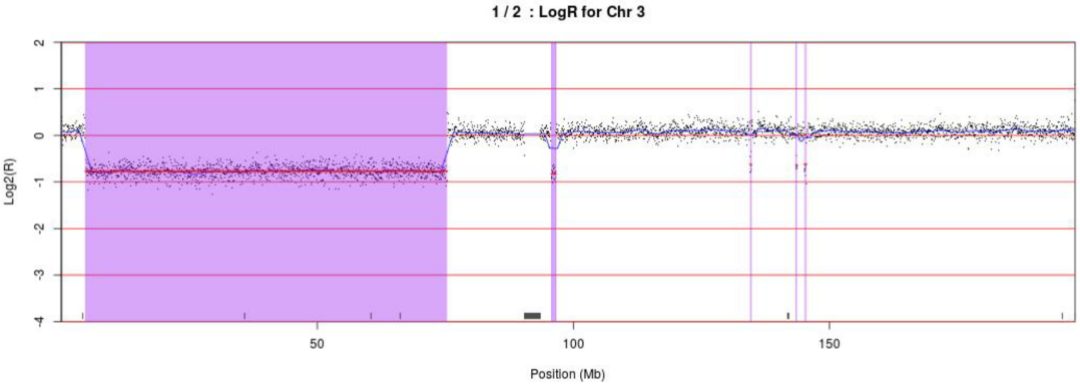

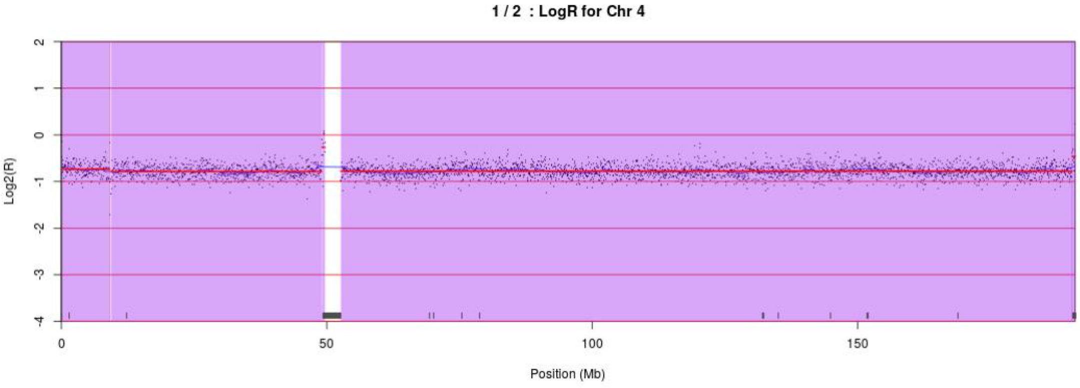

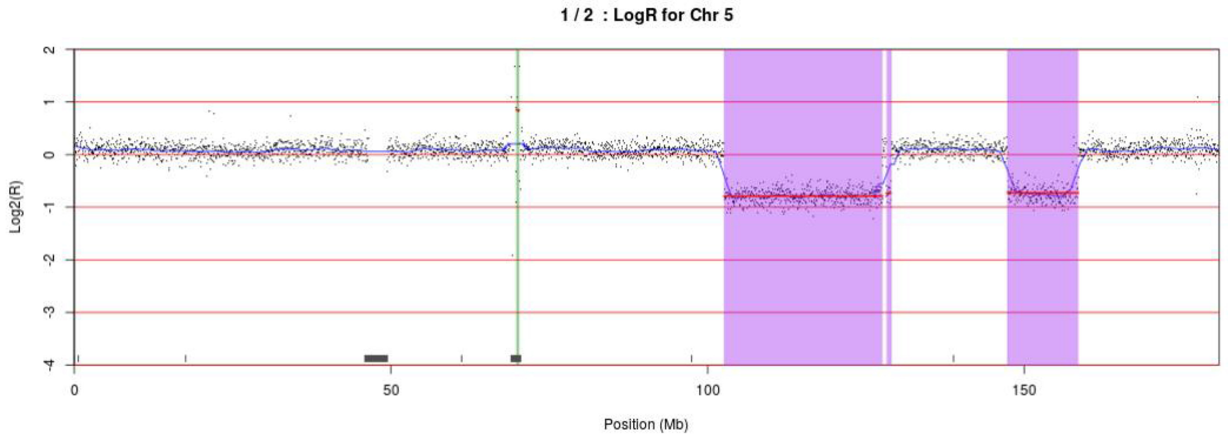
1 / 2 : LogR for Chr 5
Log2(R)
Position (Mb)

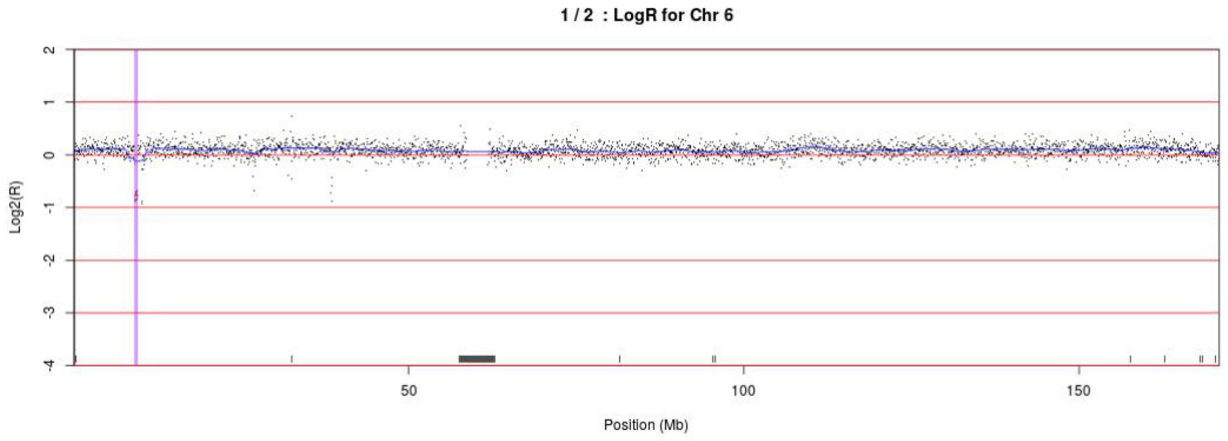
1 / 2 : LogR for Chr 6
Log2(R)
Position (Mb)

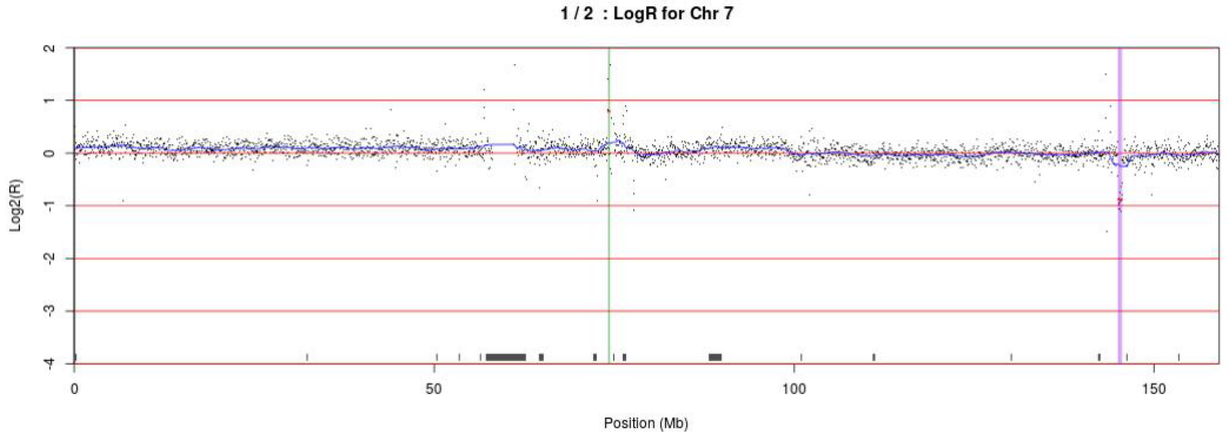
1 / 2 : LogR for Chr 7
Log2(R)
Position (Mb)

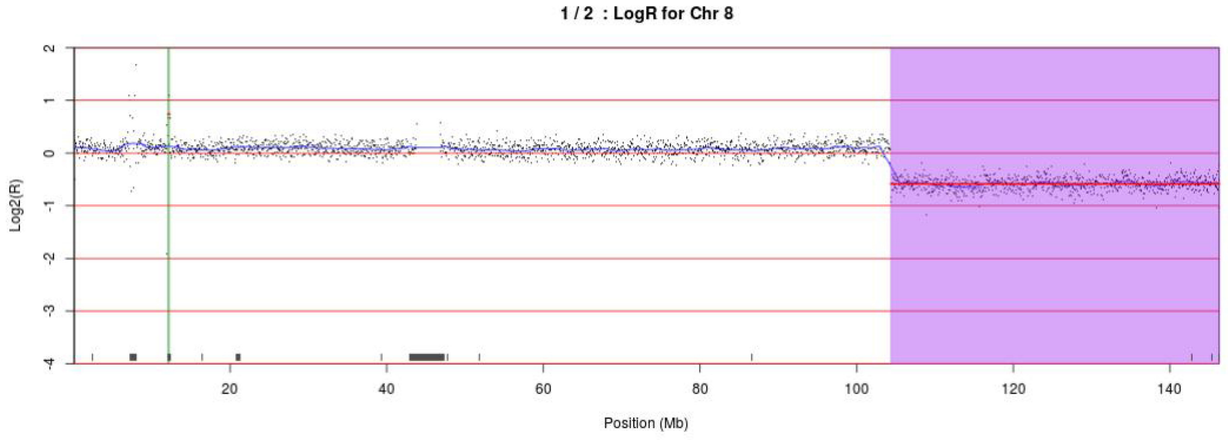
1 / 2 : LogR for Chr 8
Log2(R)
Position (Mb)

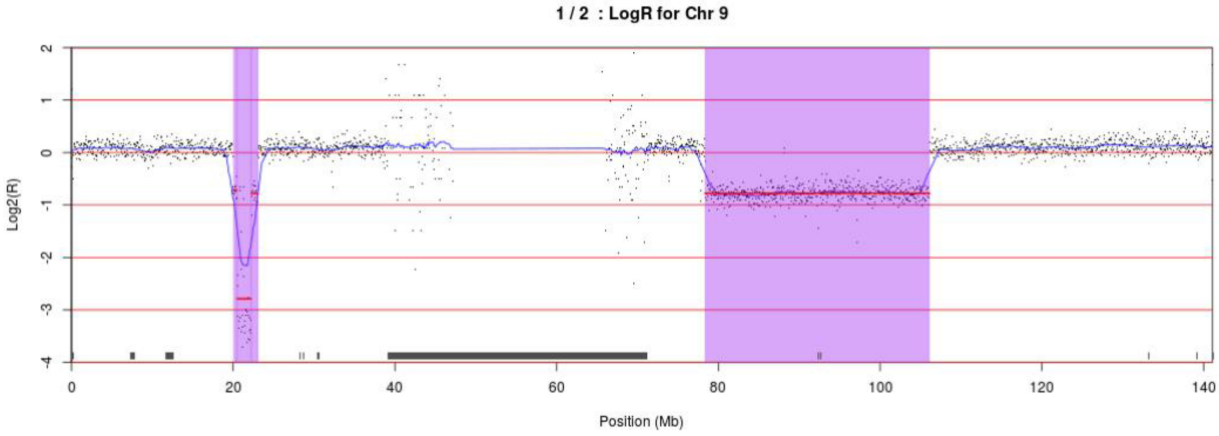
1 / 2 : LogR for Chr 9
Log2(R)
Position (Mb)

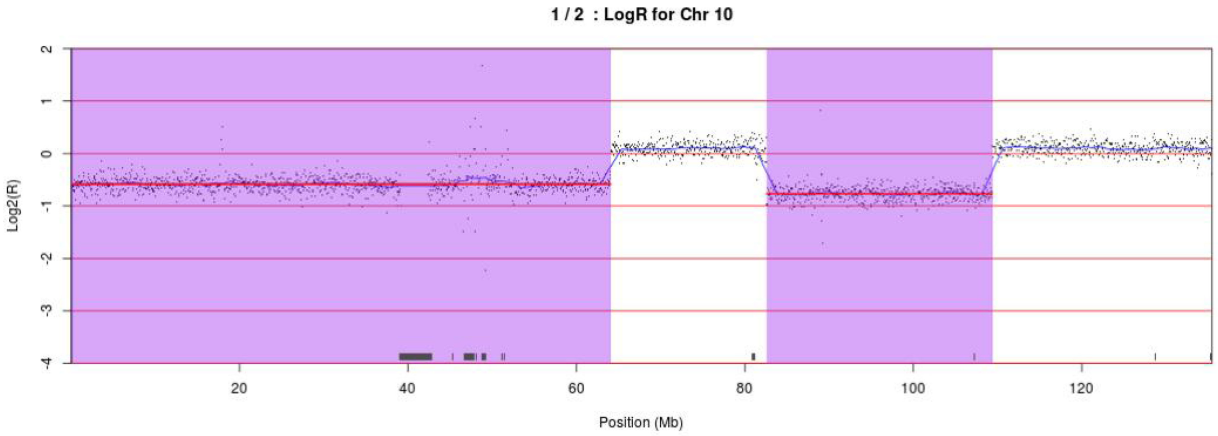
1 / 2 : LogR for Chr 10
Log2(R)
Position (Mb)

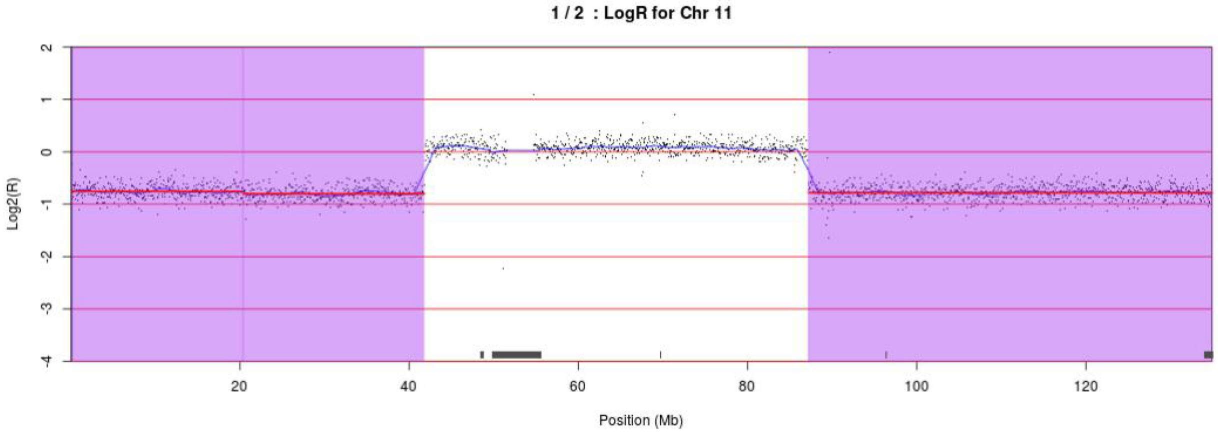
1 / 2 : LogR for Chr 11
Log2(R)
Position (Mb)

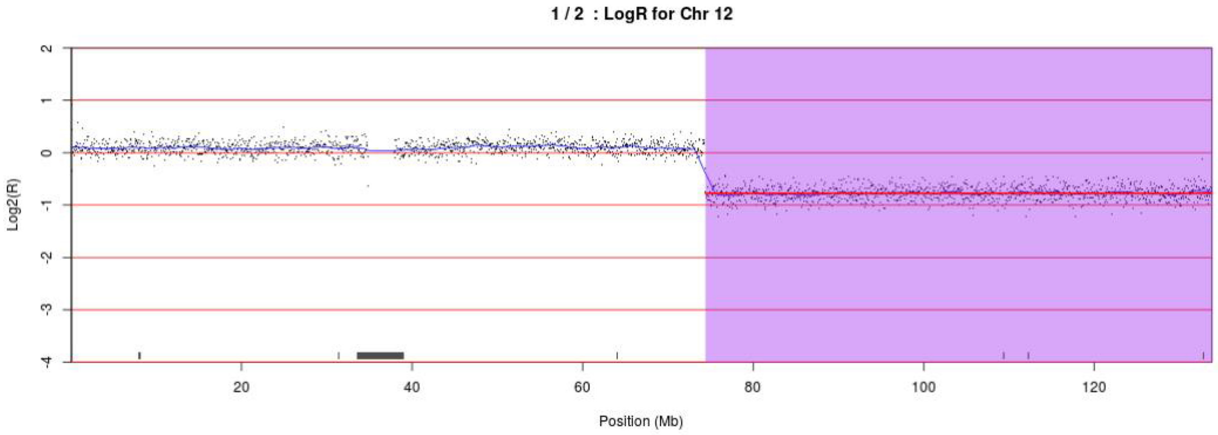
1 / 2 : LogR for Chr 12
Log2(R)
Position (Mb)

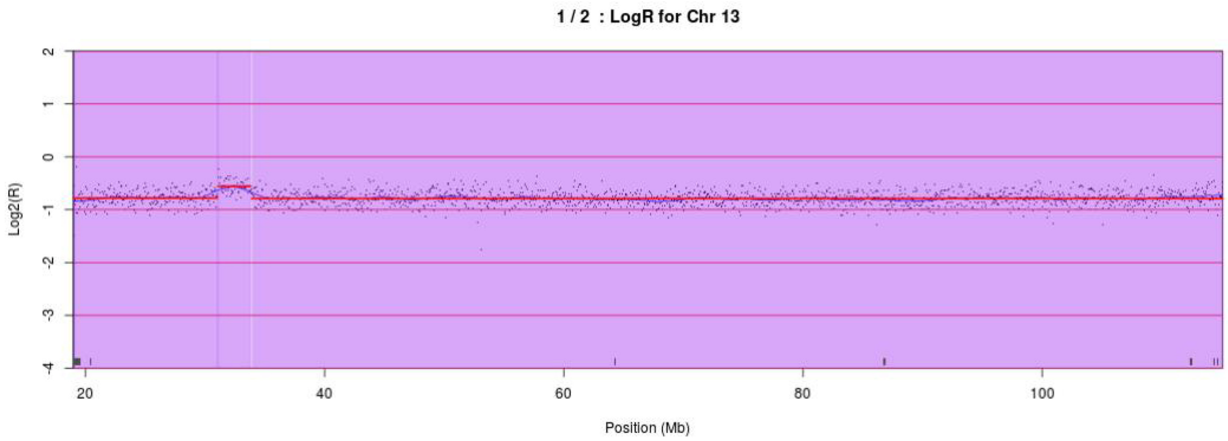
1 / 2 : LogR for Chr 13
Log2(R)
Position (Mb)

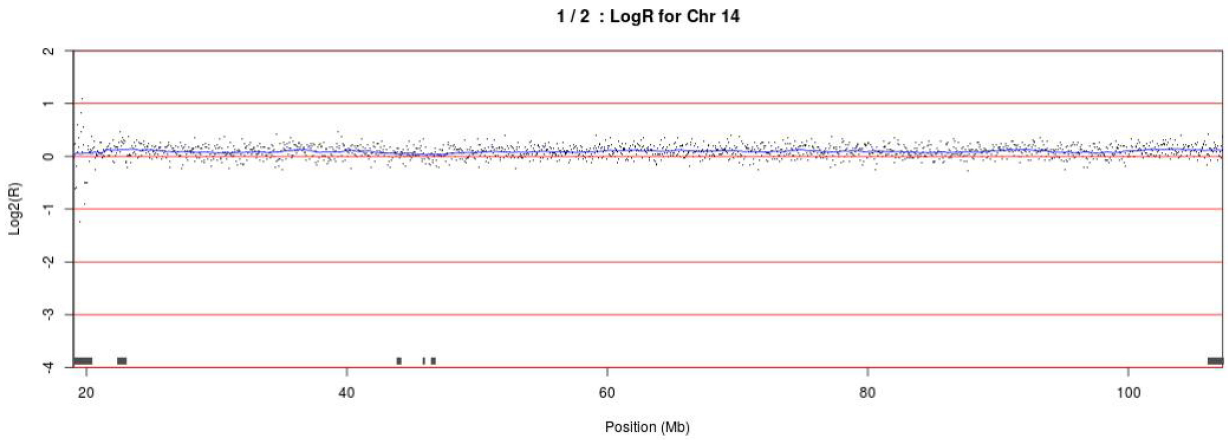
1 / 2 : LogR for Chr 14
Log2(R)
Position (Mb)

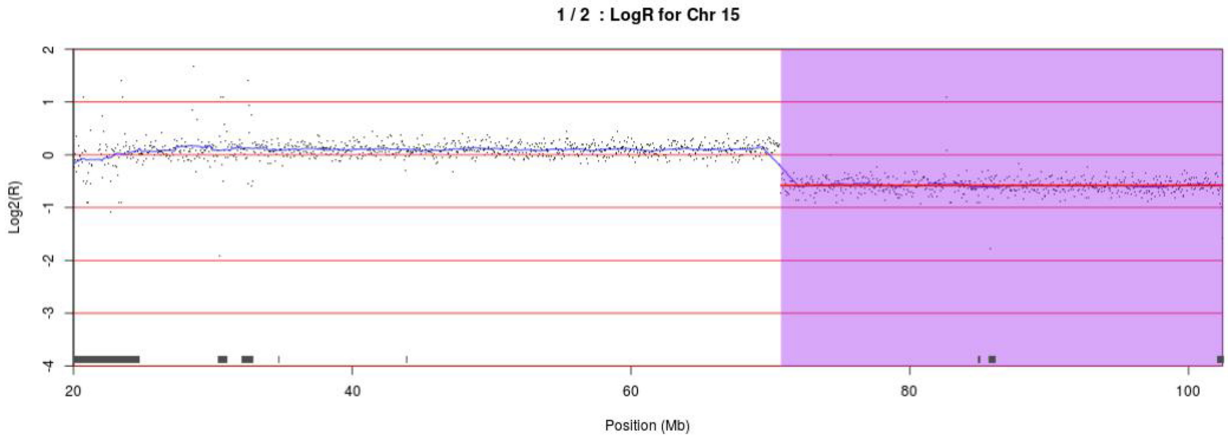
1 / 2 : LogR for Chr 15
Log2(R)
Position (Mb)

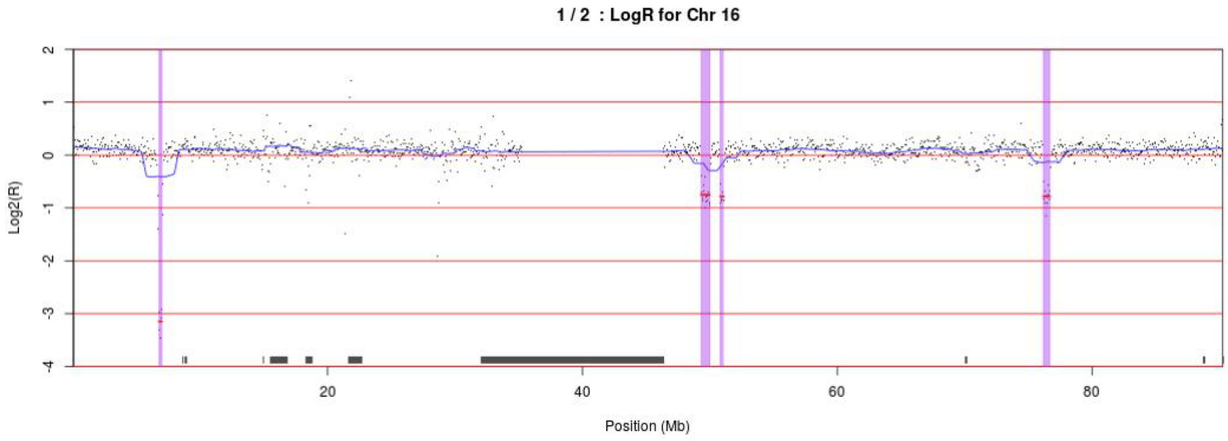
1 / 2 : LogR for Chr 16
Log2(R)
Position (Mb)

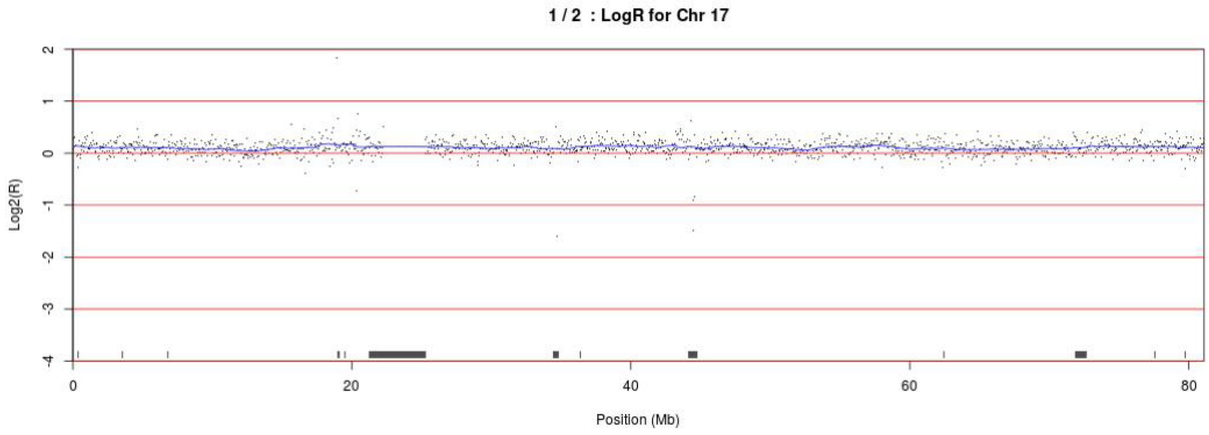
1 / 2 : LogR for Chr 17
Log2(R)
Position (Mb)

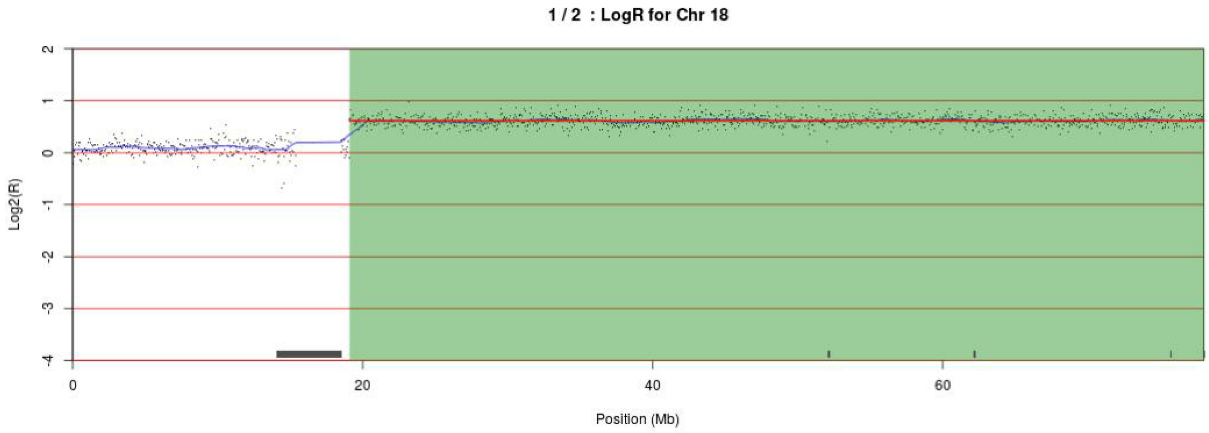
1 / 2 : LogR for Chr 18
Log2(R)
Position (Mb)

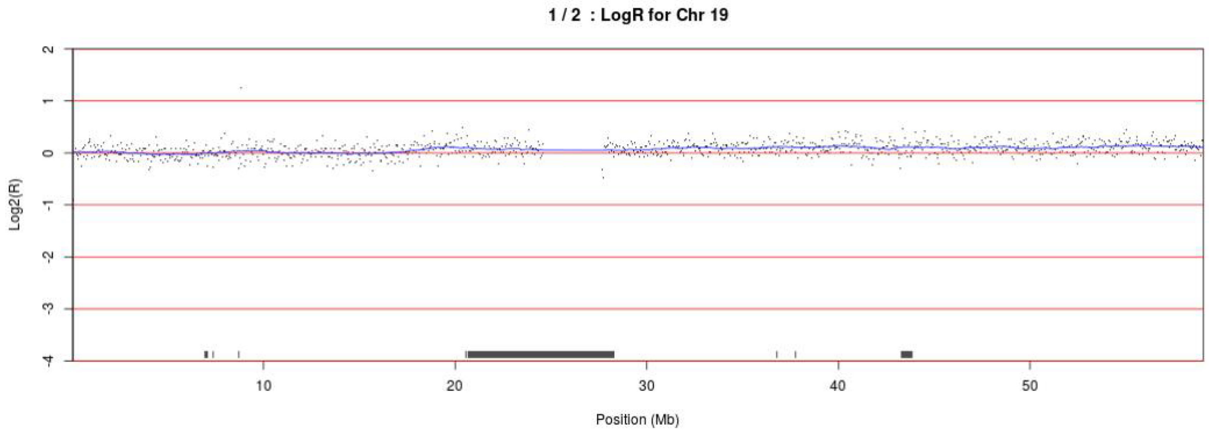
1 / 2 : LogR for Chr 19
Log2(R)
Position (Mb)

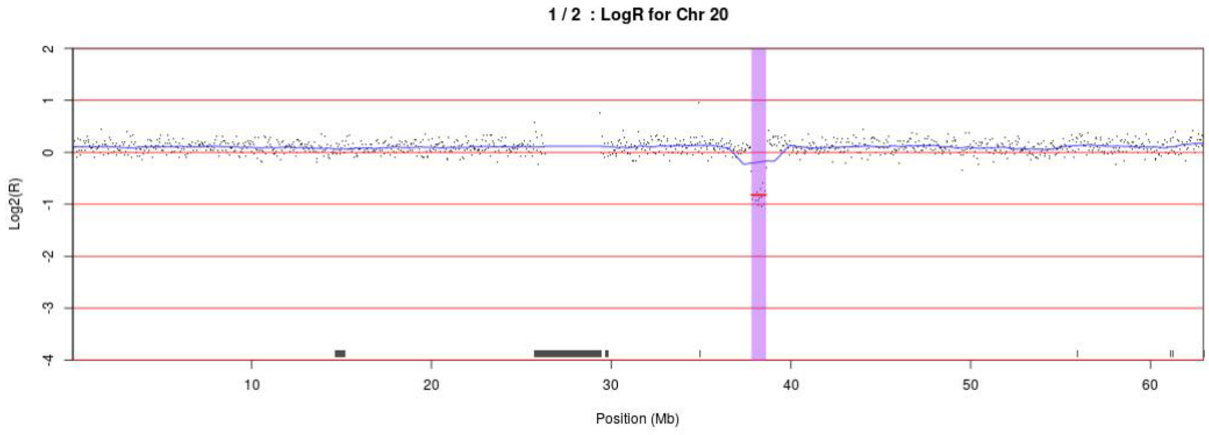
1 / 2 : LogR for Chr 20
Log2(R)
Position (Mb)

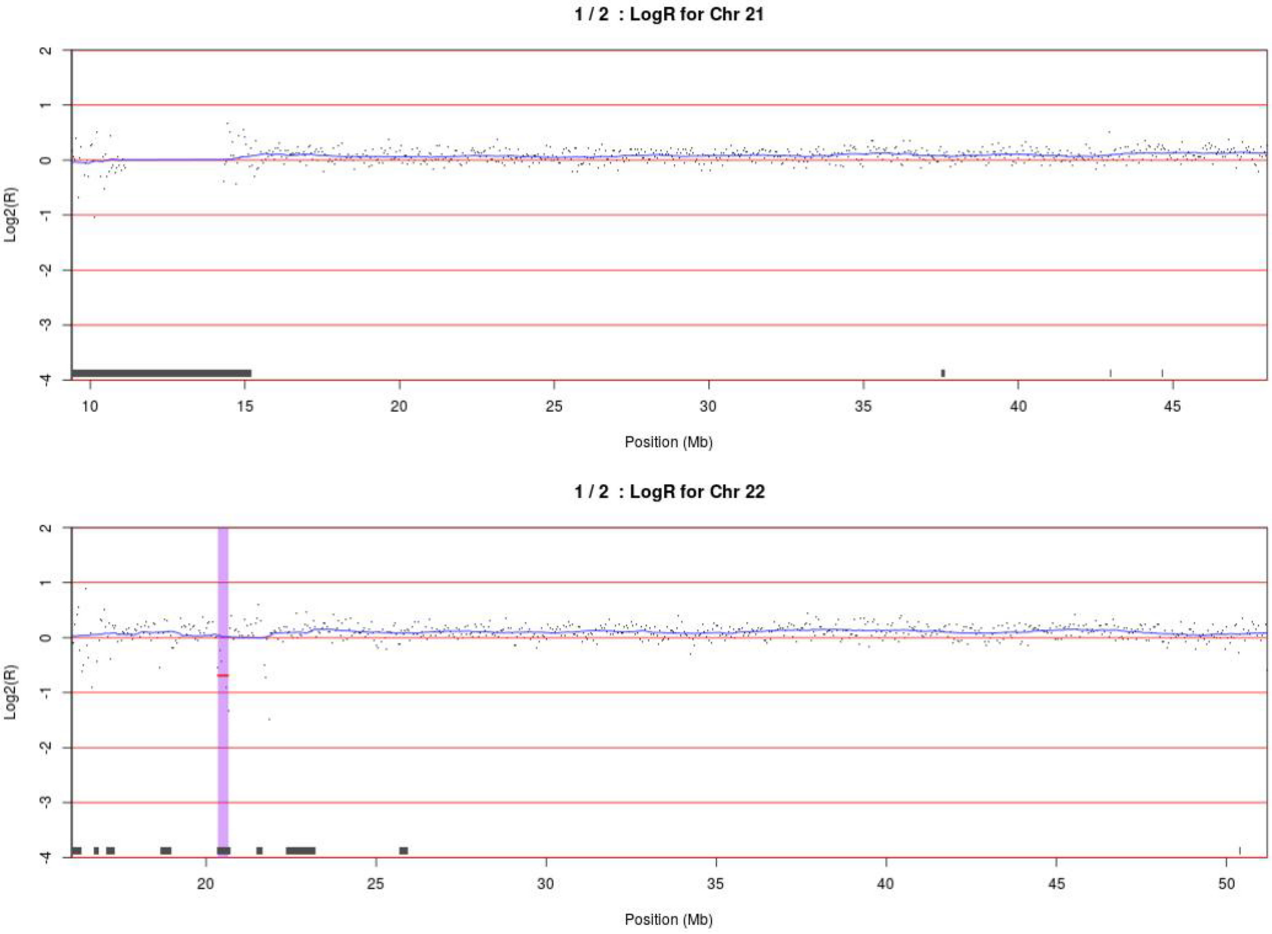

Supplementary Figure 1: Copy number profile of a representative tumor/normal sample pair. Log_2-ratios were plotted against chromosome position.

Chr1

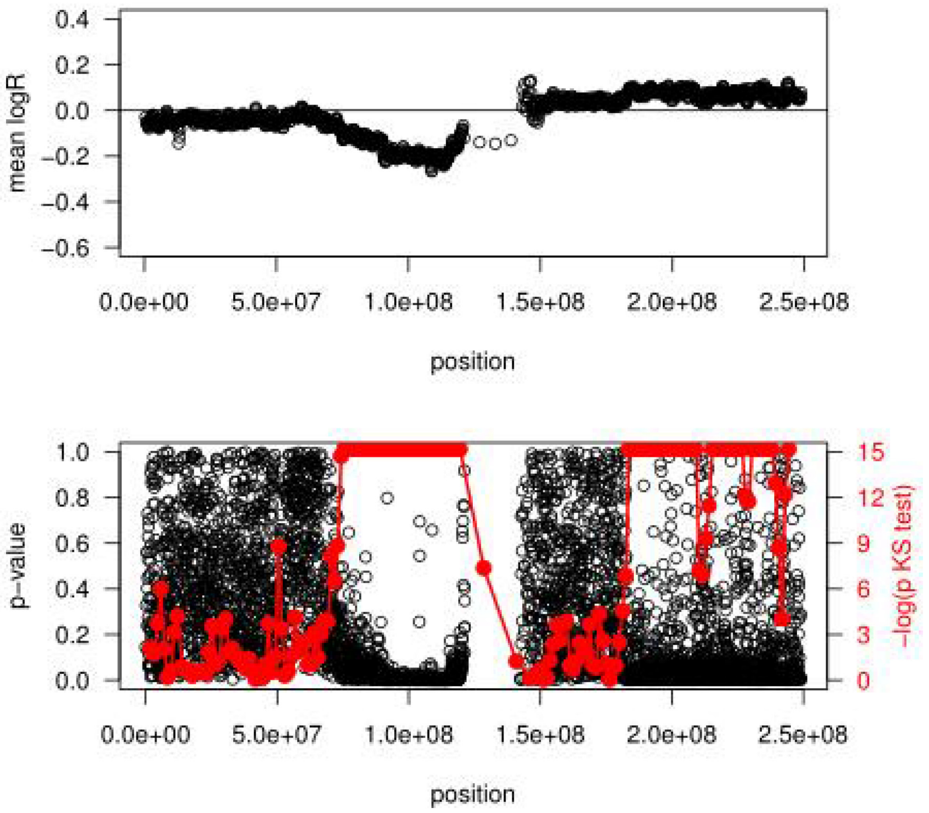
mean logR
0.4
0.2
0.0
−0.2
−0.4
−0.6
0.0e+00
5.0e+07
1.0e+08
1.5e+08
2.0e+08
2.5e+08
position
p−value
1.0
0.8
0.6
0.4
0.2
0.0
−log(p KS test)
15
12
9
6
3
0
position

Chr2

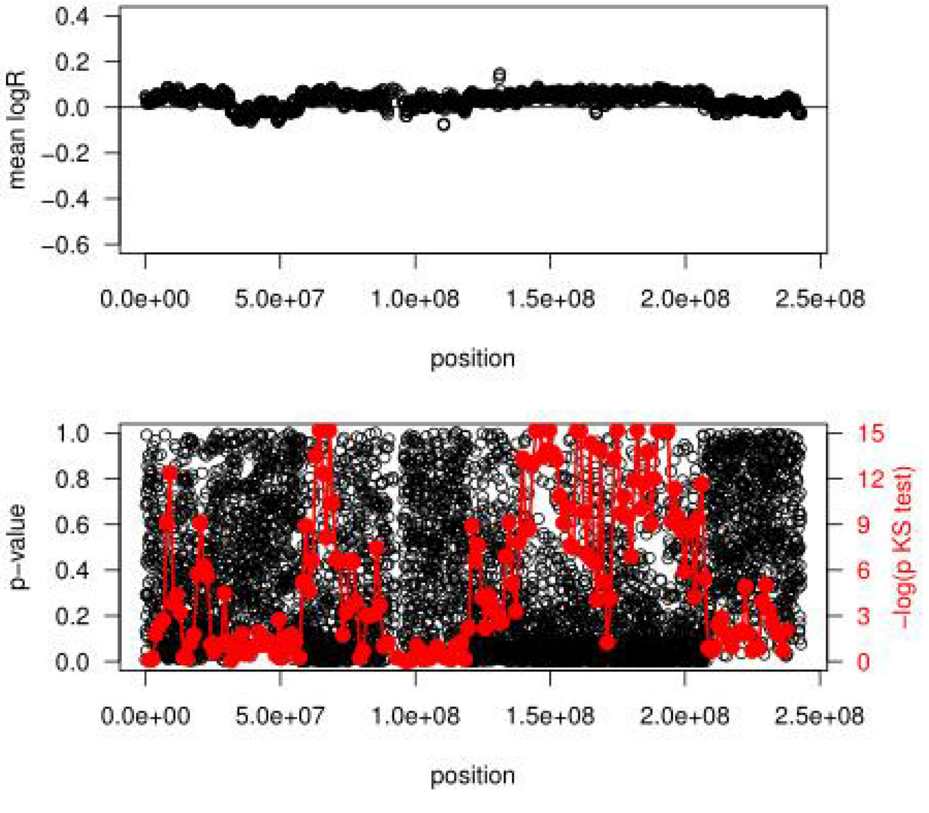
mean logR
0.4
0.2
0.0
−0.2
−0.4
−0.6
0.0e+00
5.0e+07
1.0e+08
1.5e+08
2.0e+08
2.5e+08
position
p−value
1.0
0.8
0.6
0.4
0.2
0.0
−log(p KS test)
15
12
9
6
3
0
position

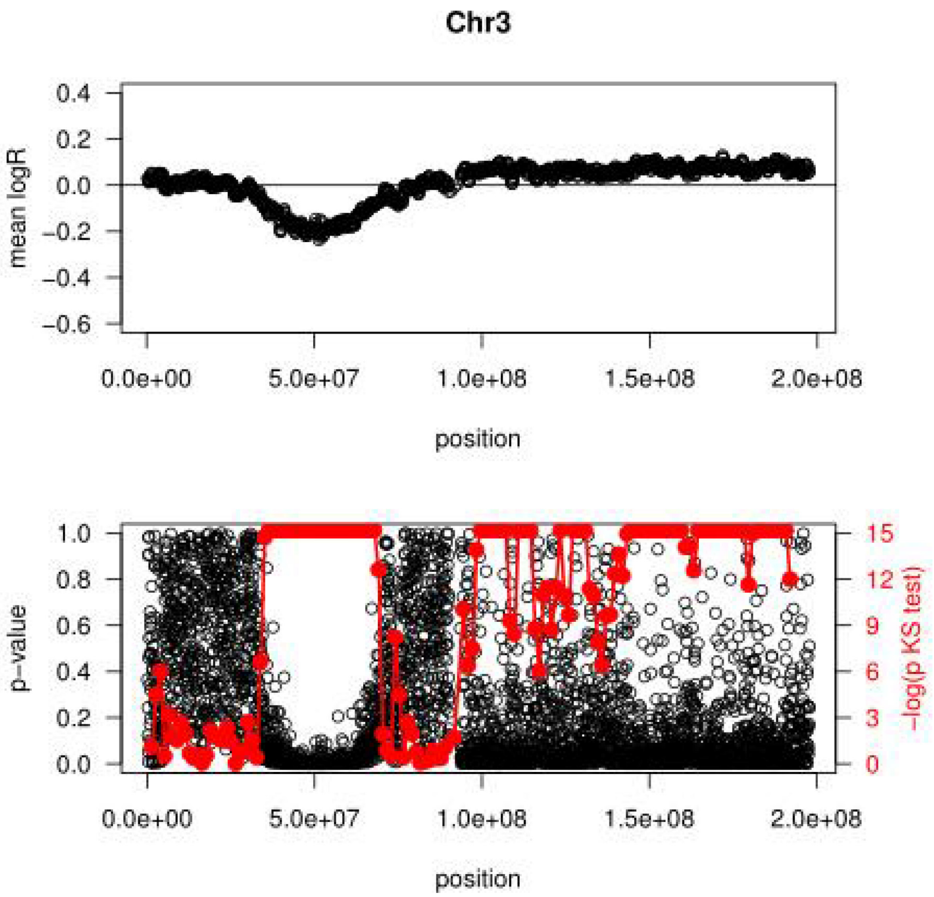
Chr3
mean logR
0.4
0.2
0.0
−0.2
−0.4
−0.6
0.0e+00
5.0e+07
1.0e+08
1.5e+08
2.0e+08
position
p−value
1.0
0.8
0.6
0.4
0.2
0.0
−log(p KS test)
15
12
9
6
3
0
position

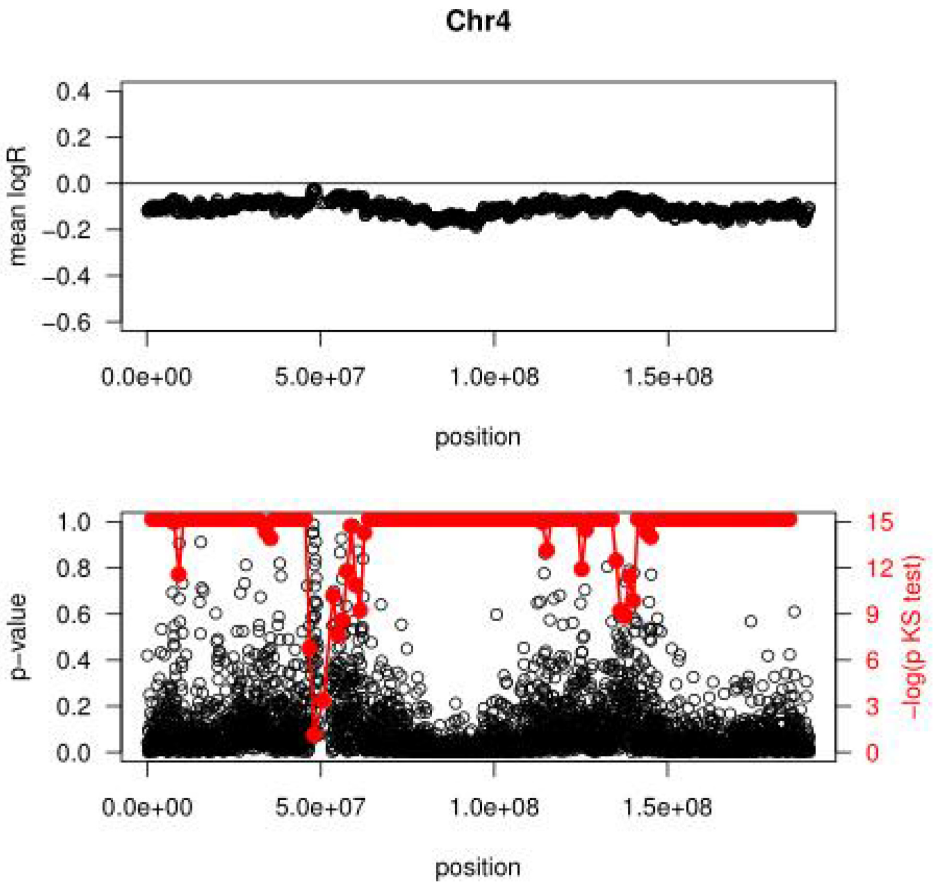
Chr4
mean logR
0.4
0.2
0.0
−0.2
−0.4
−0.6
0.0e+00
5.0e+07
1.0e+08
1.5e+08
position
p−value
1.0
0.8
0.6
0.4
0.2
0.0
−log(p KS test)
15
12
9
6
3
0
position

Chr5

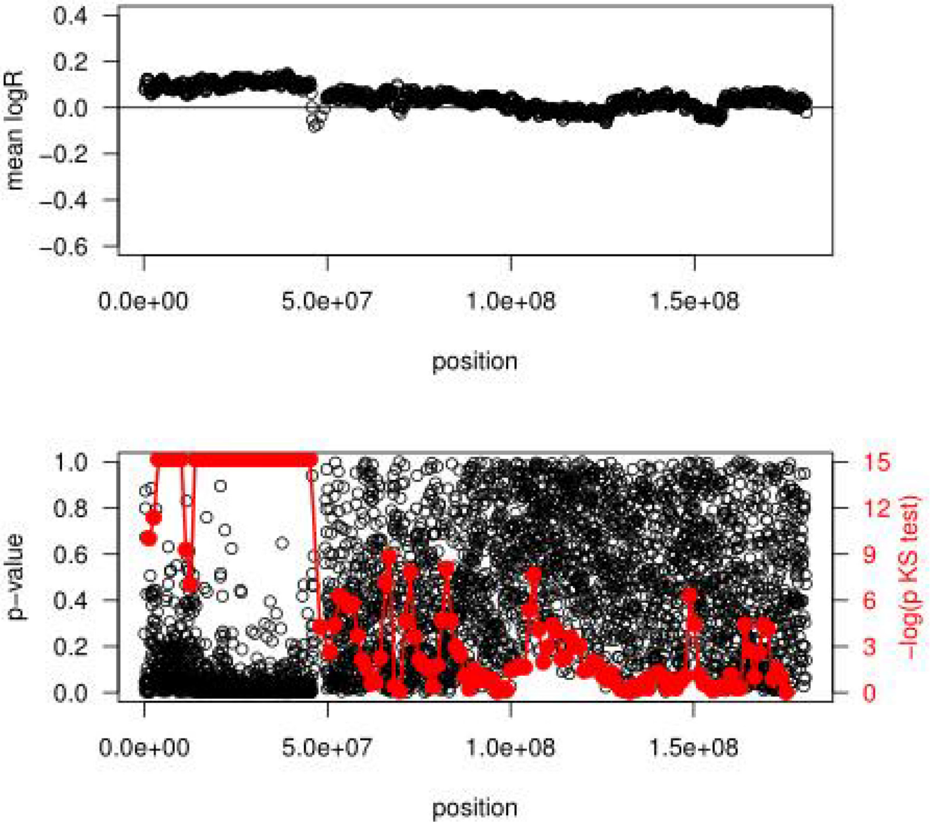
mean logR
0.4
0.2
0.0
−0.2
−0.4
−0.6
0.0e+00
5.0e+07
1.0e+08
1.5e+08
position
p−value
1.0
0.8
0.6
0.4
0.2
0.0
−log(p KS test)
15
12
9
6
3
0
0.0e+00
5.0e+07
1.0e+08
1.5e+08
position

Chr6

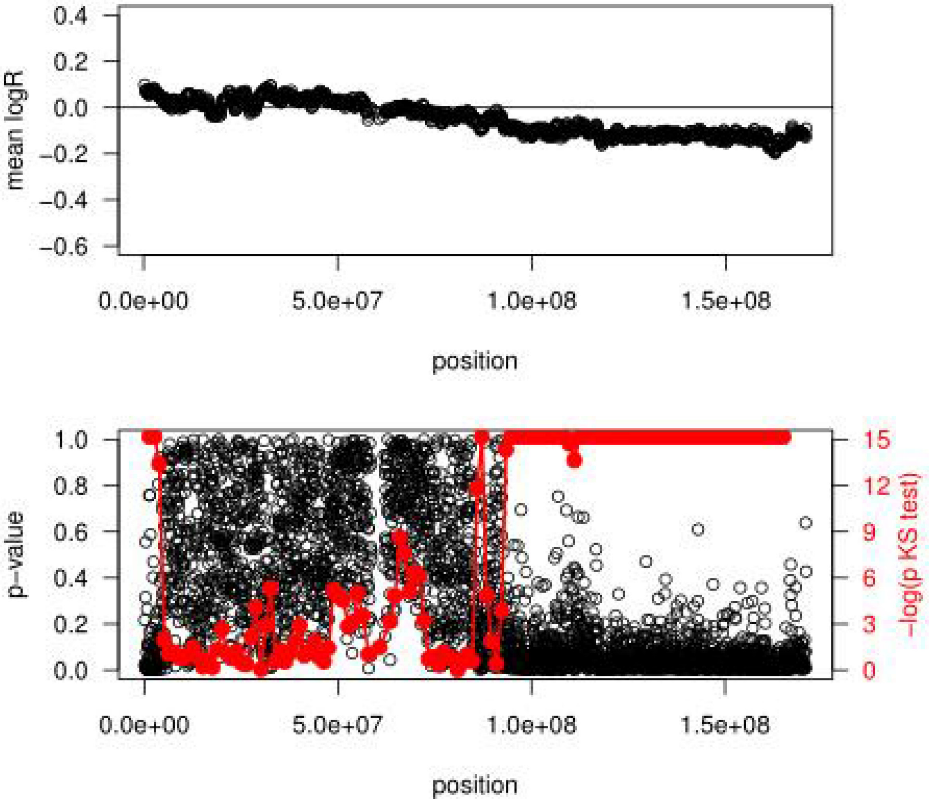
mean logR
0.4
0.2
0.0
−0.2
−0.4
−0.6
0.0e+00
5.0e+07
1.0e+08
1.5e+08
position
p−value
1.0
0.8
0.6
0.4
0.2
0.0
−log(p KS test)
15
12
9
6
3
0
0.0e+00
5.0e+07
1.0e+08
1.5e+08
position

Chr7

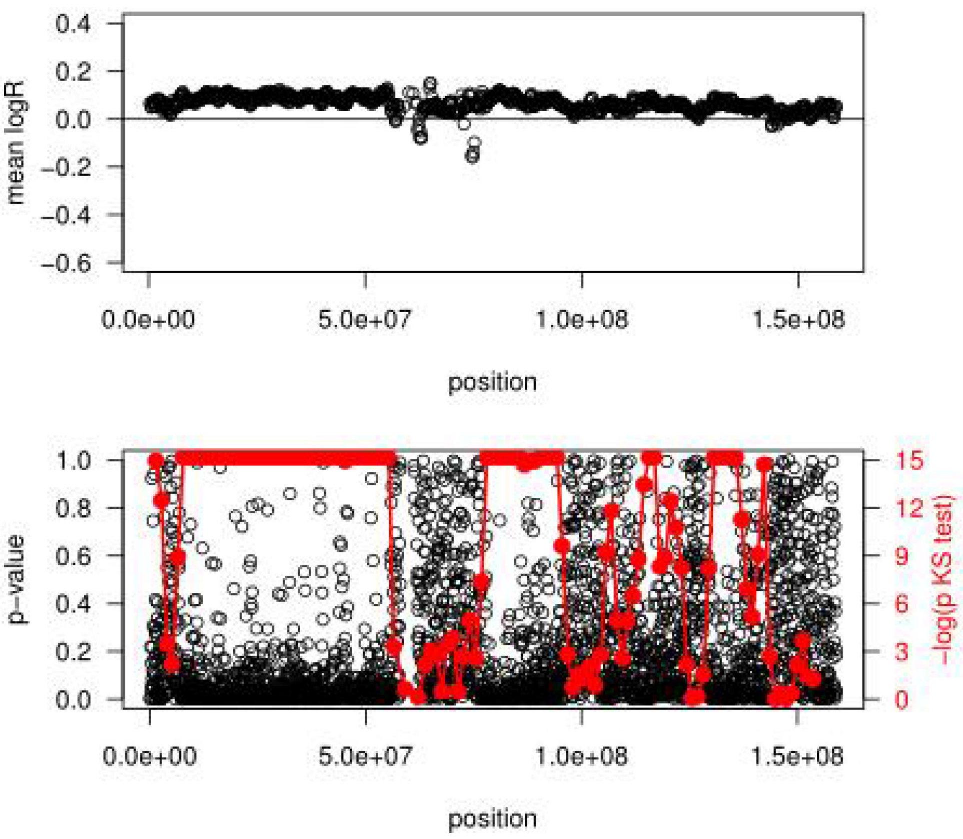
mean logR
position
p−value
−log(p KS test)
position

Chr8

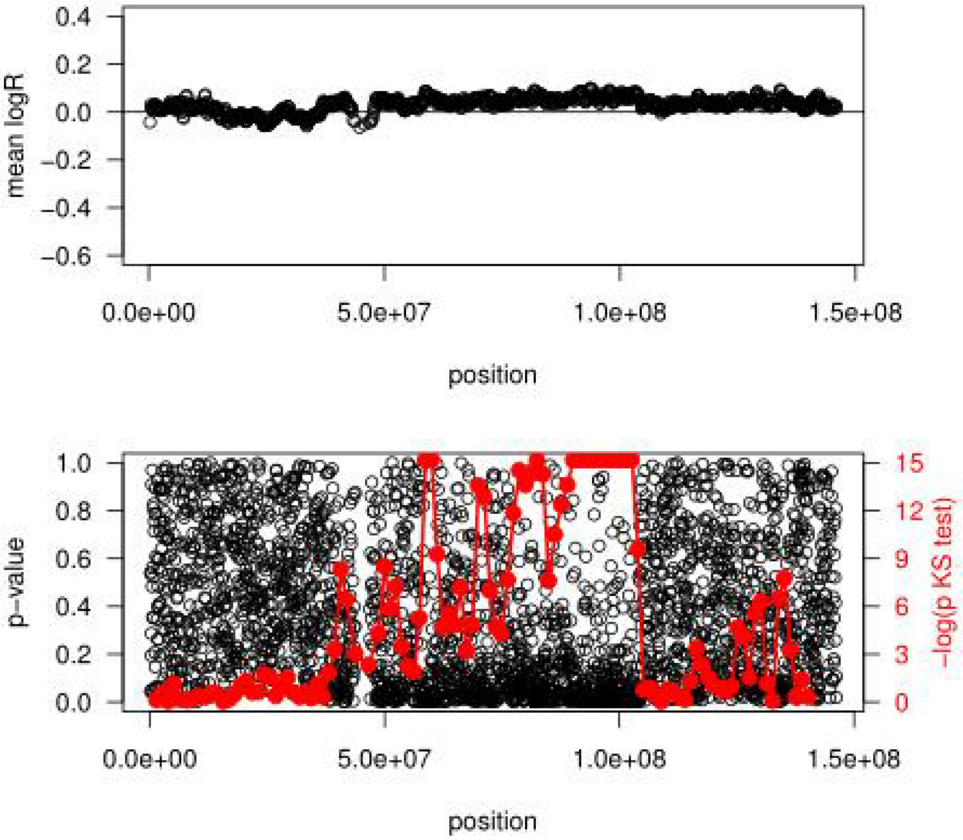
mean logR
position
p−value
−log(p KS test)
position

Chr9

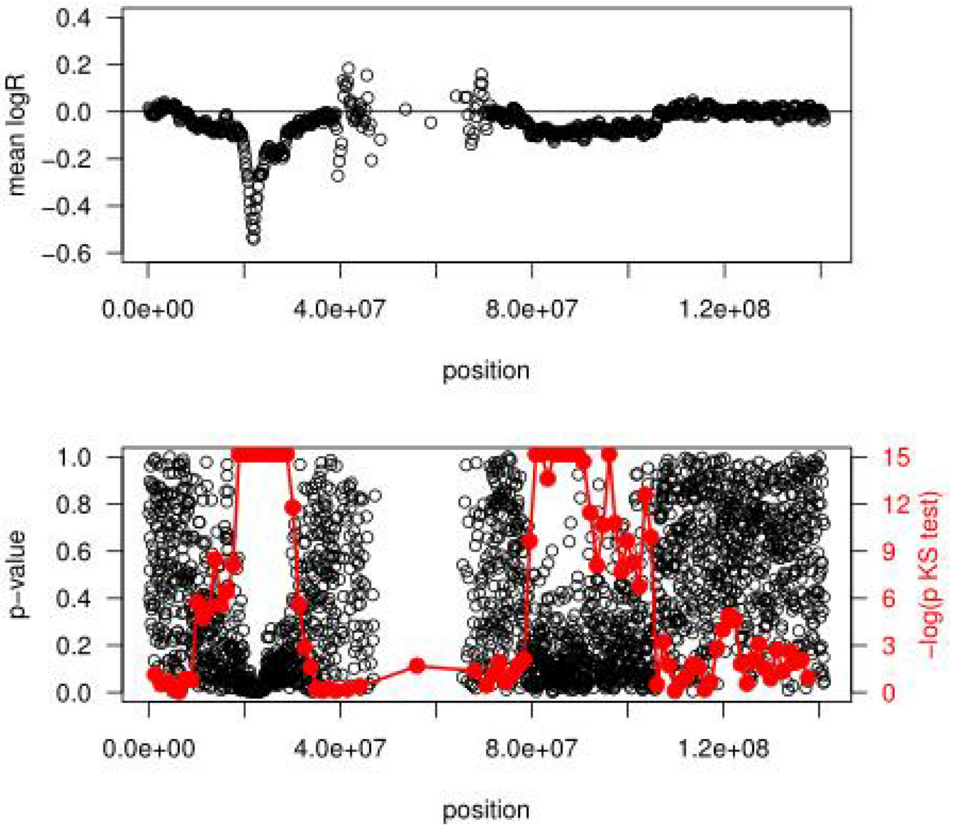
0.4
0.2
0.0
−0.2
−0.4
−0.6
mean logR
0.0e+00
4.0e+07
8.0e+07
1.2e+08
position
1.0
0.8
0.6
0.4
0.2
0.0
p−value
15
12
9
6
3
0
−log(p KS test)
0.0e+00
4.0e+07
8.0e+07
1.2e+08
position

Chr10

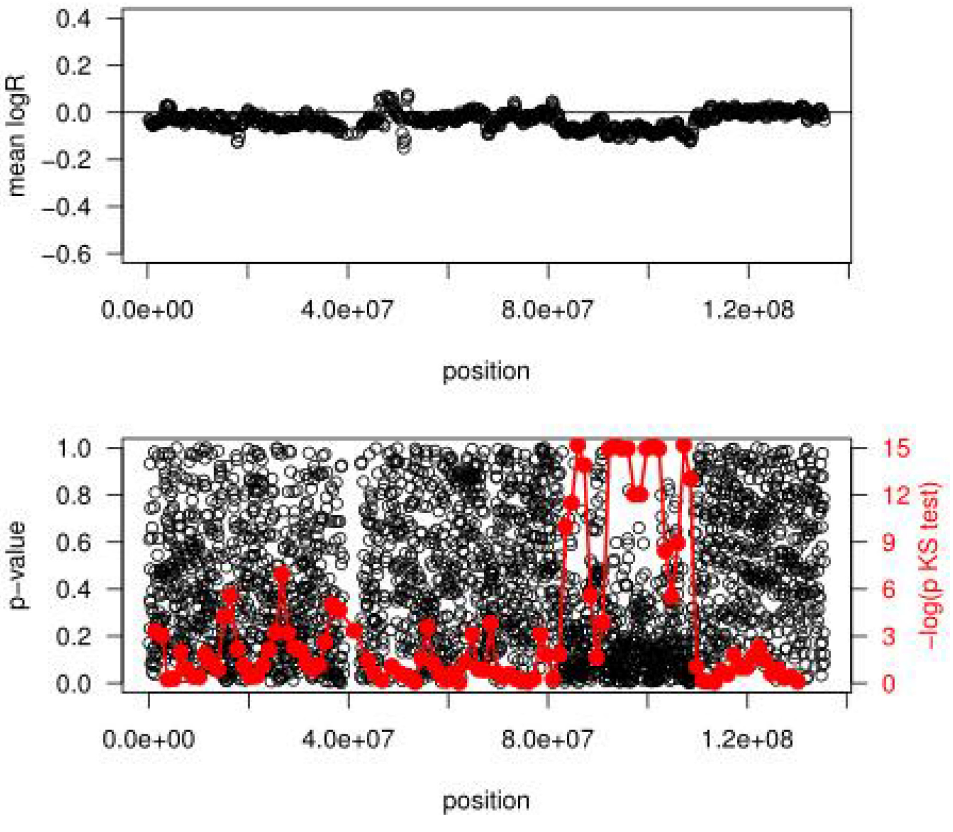
0.4
0.2
0.0
−0.2
−0.4
−0.6
mean logR
0.0e+00
4.0e+07
8.0e+07
1.2e+08
position
1.0
0.8
0.6
0.4
0.2
0.0
p−value
15
12
9
6
3
0
−log(p KS test)
0.0e+00
4.0e+07
8.0e+07
1.2e+08
position

Chr11

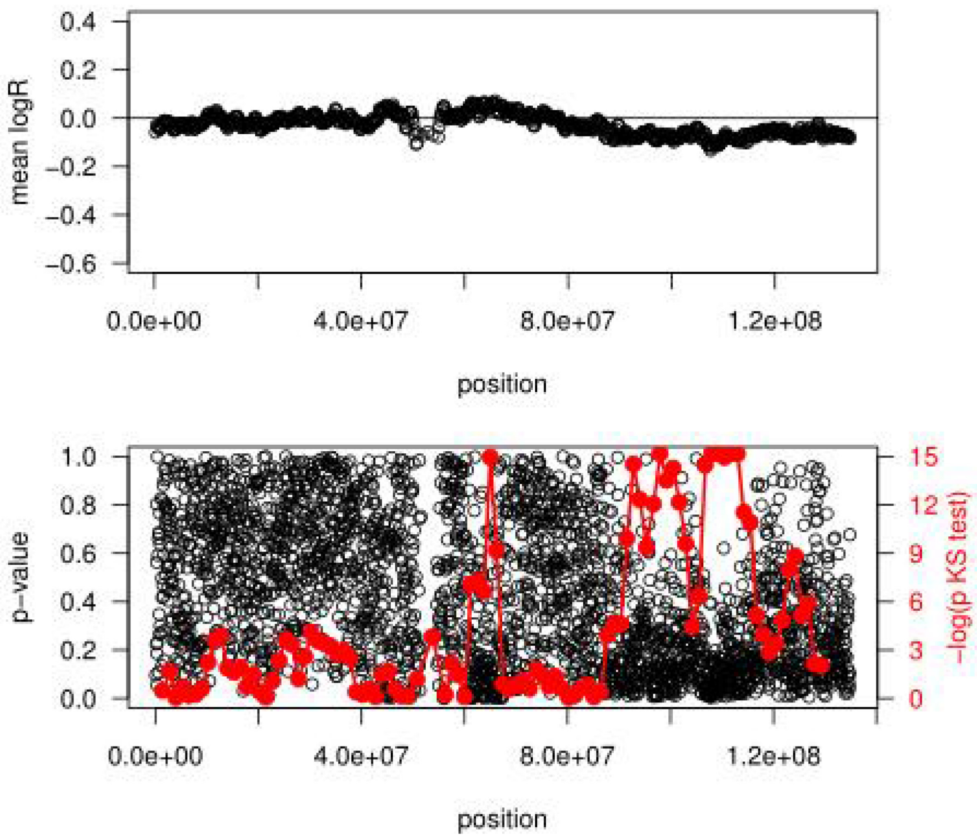
mean logR
0.4
0.2
0.0
−0.2
−0.4
−0.6
0.0e+00
4.0e+07
8.0e+07
1.2e+08
position
p−value
1.0
0.8
0.6
0.4
0.2
0.0
15
12
9
6
3
0
−log(p KS test)
position

Chr12

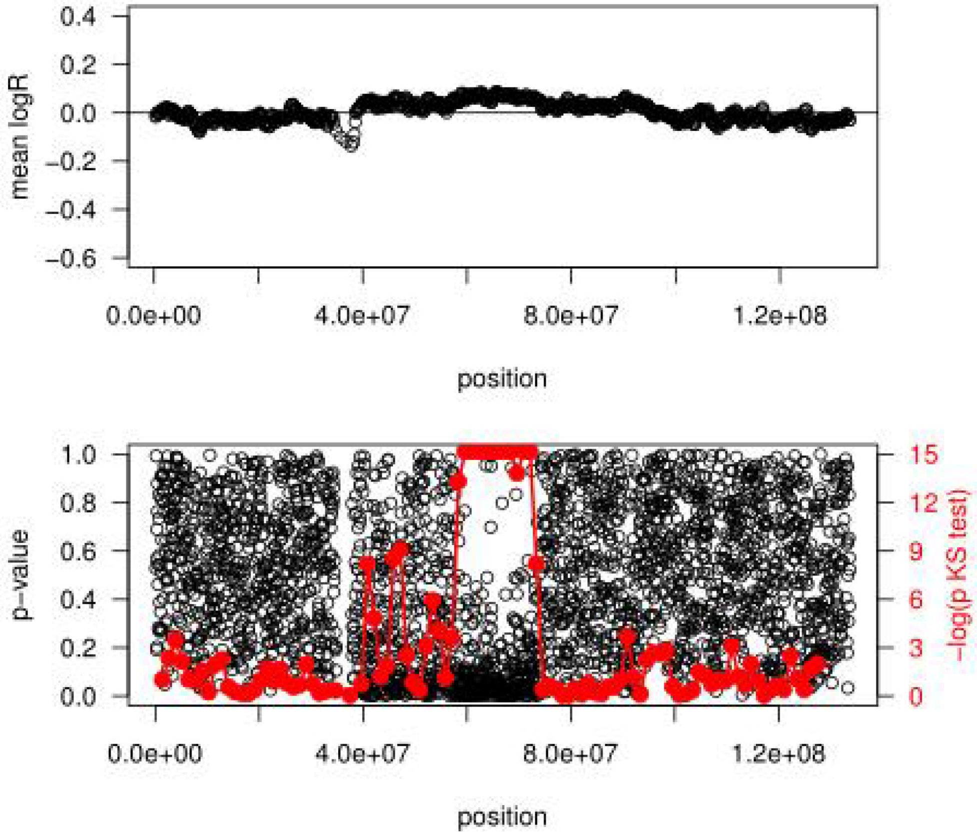
mean logR
0.4
0.2
0.0
−0.2
−0.4
−0.6
0.0e+00
4.0e+07
8.0e+07
1.2e+08
position
p−value
1.0
0.8
0.6
0.4
0.2
0.0
15
12
9
6
3
0
−log(p KS test)
position

Chr13

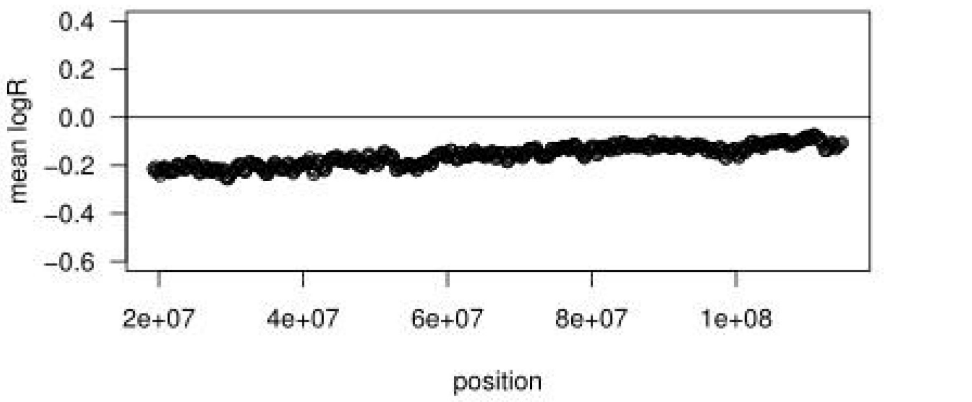
mean logR
0.4
0.2
0.0
−0.2
−0.4
−0.6
2e+07
4e+07
6e+07
8e+07
1e+08
position
p−value
1.0
0.8
0.6
0.4
0.2
0.0
−log(p KS test)
15
12
9
6
3
0
position

Chr14

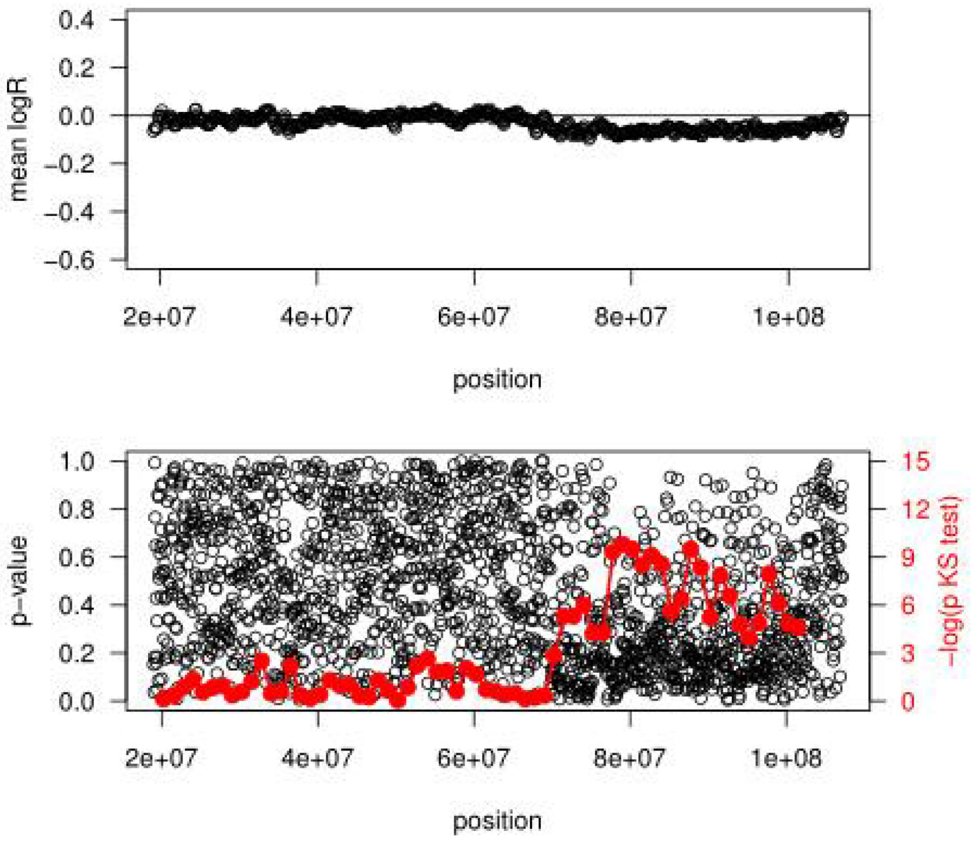
mean logR
0.4
0.2
0.0
−0.2
−0.4
−0.6
2e+07
4e+07
6e+07
8e+07
1e+08
position
p−value
1.0
0.8
0.6
0.4
0.2
0.0
−log(p KS test)
15
12
9
6
3
0
position

Chr15

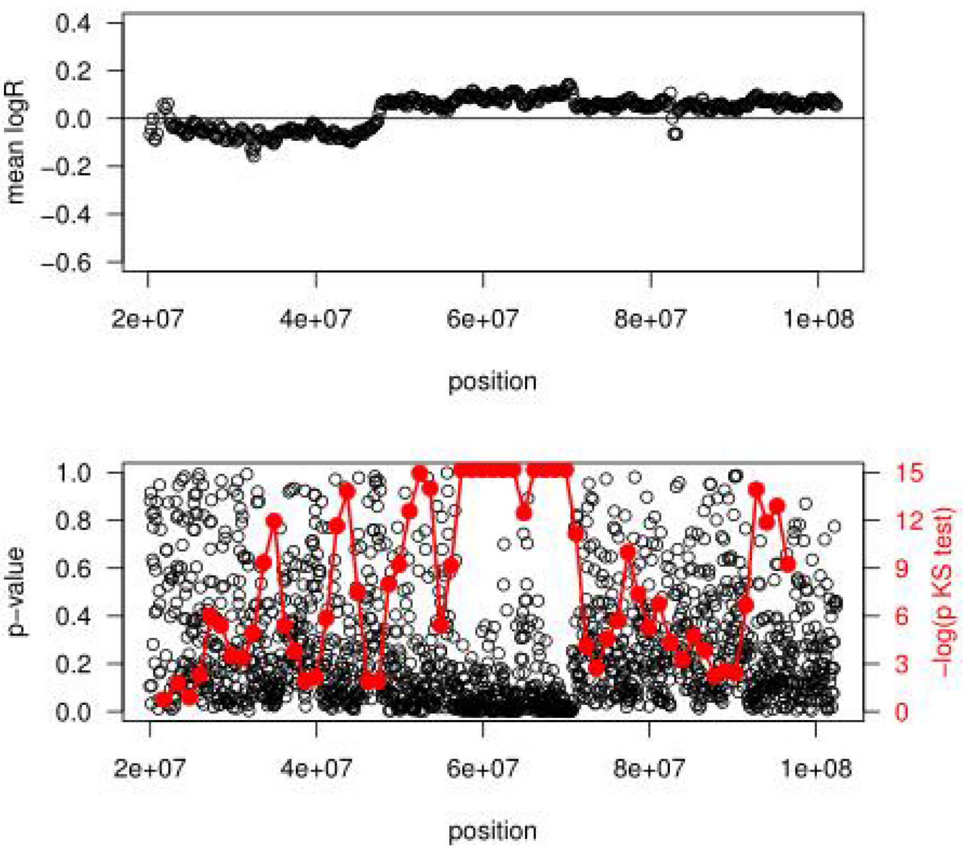
mean logR
position
p−value
−log(p KS test)
position

Chr16

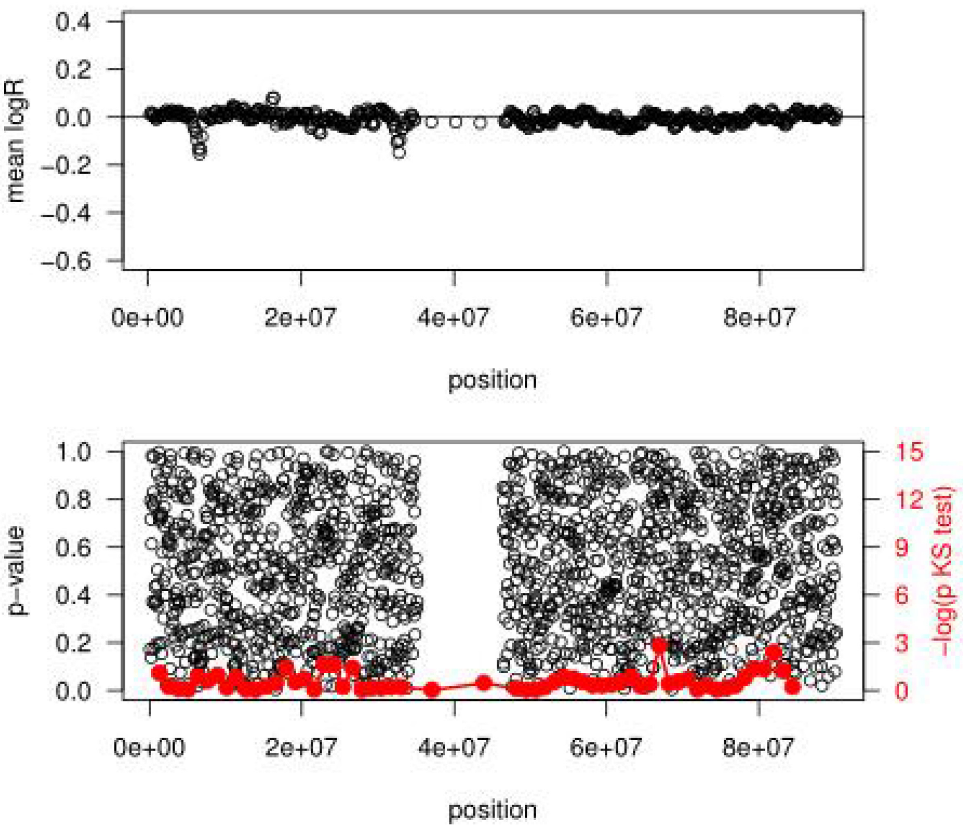
mean logR
position
p−value
−log(p KS test)
position

Chr17

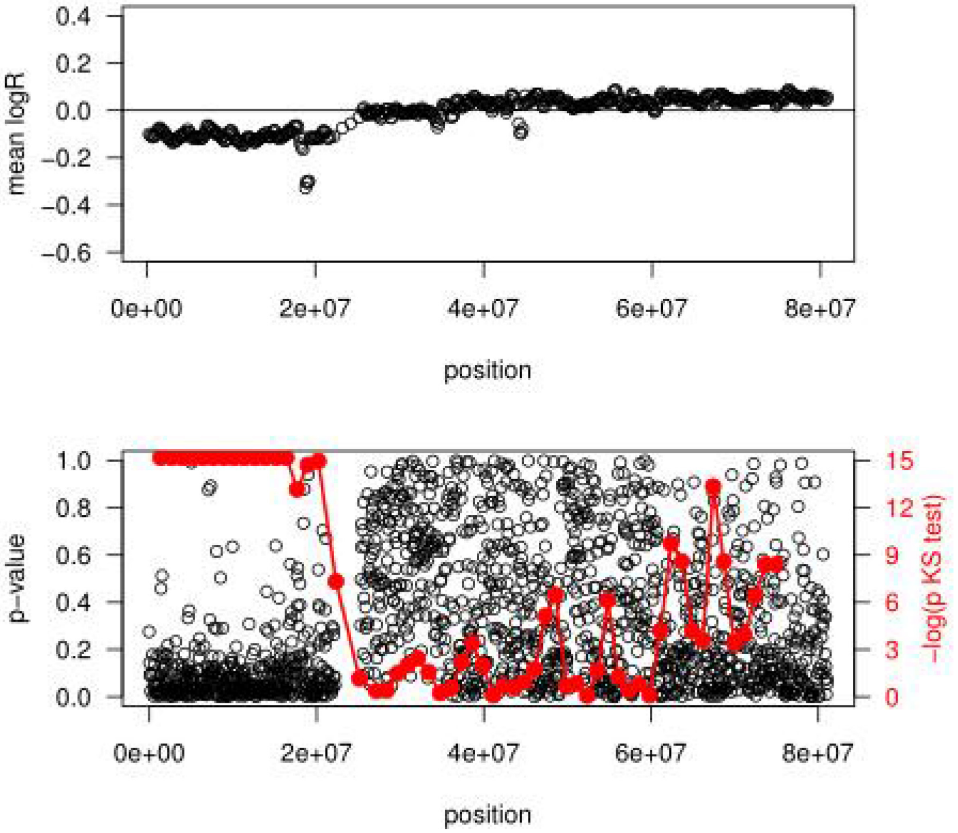
mean logR
position
p−value
−log(p KS test)
0e+00
2e+07
4e+07
6e+07
8e+07

Chr18

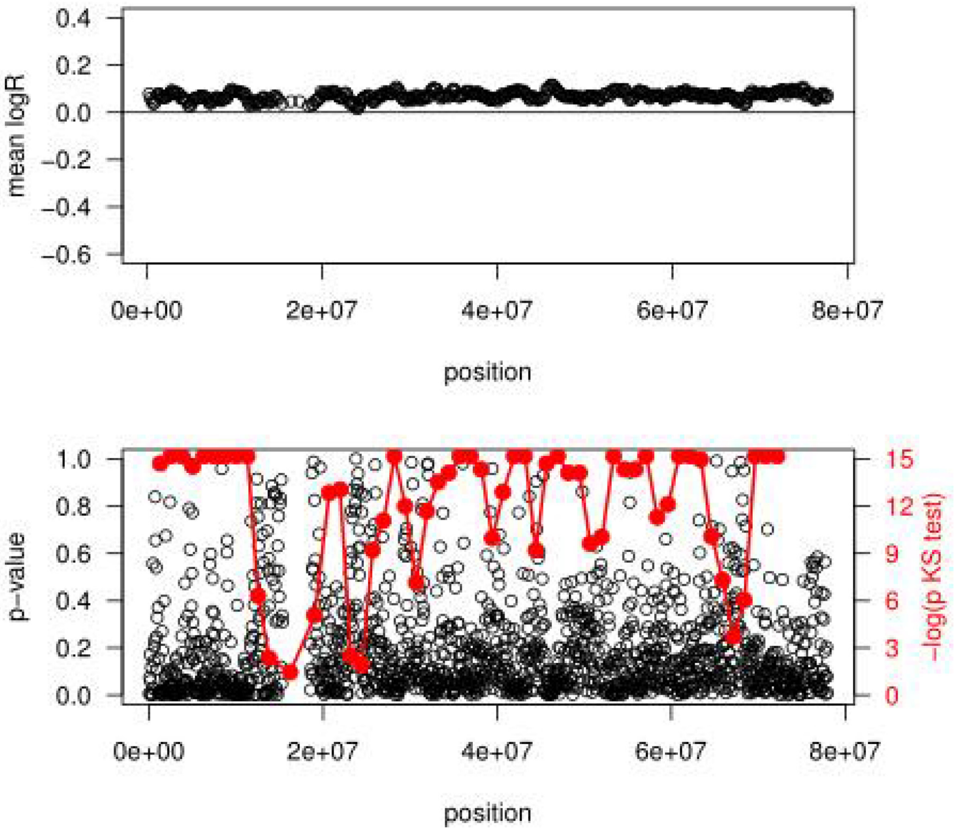
mean logR
position
p−value
−log(p KS test)
0e+00
2e+07
4e+07
6e+07
8e+07

Chr19

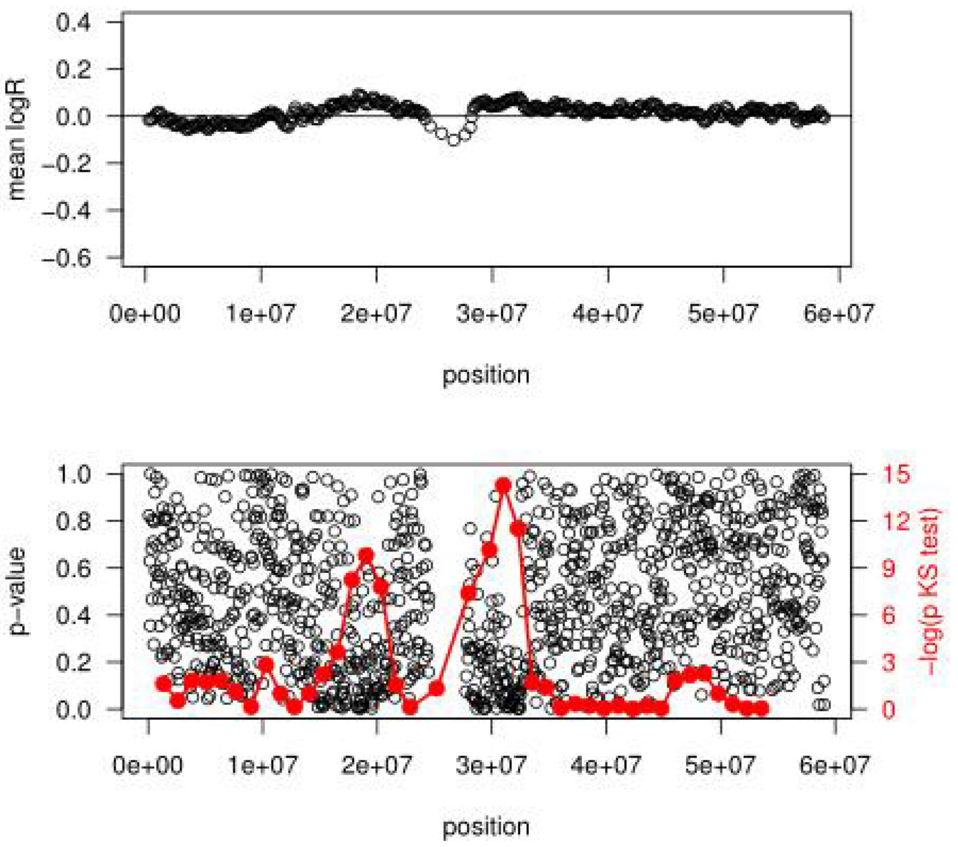
mean logR
position
p−value
−log(p KS test)
position

Chr20

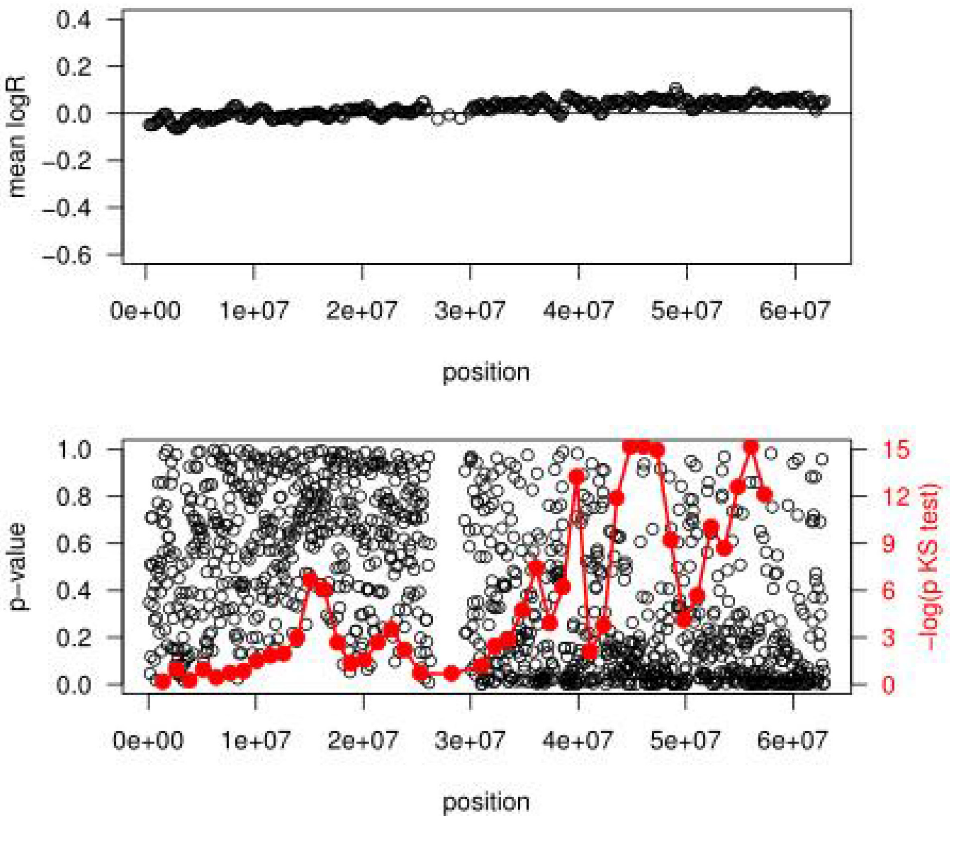
mean logR
position
p−value
−log(p KS test)
position

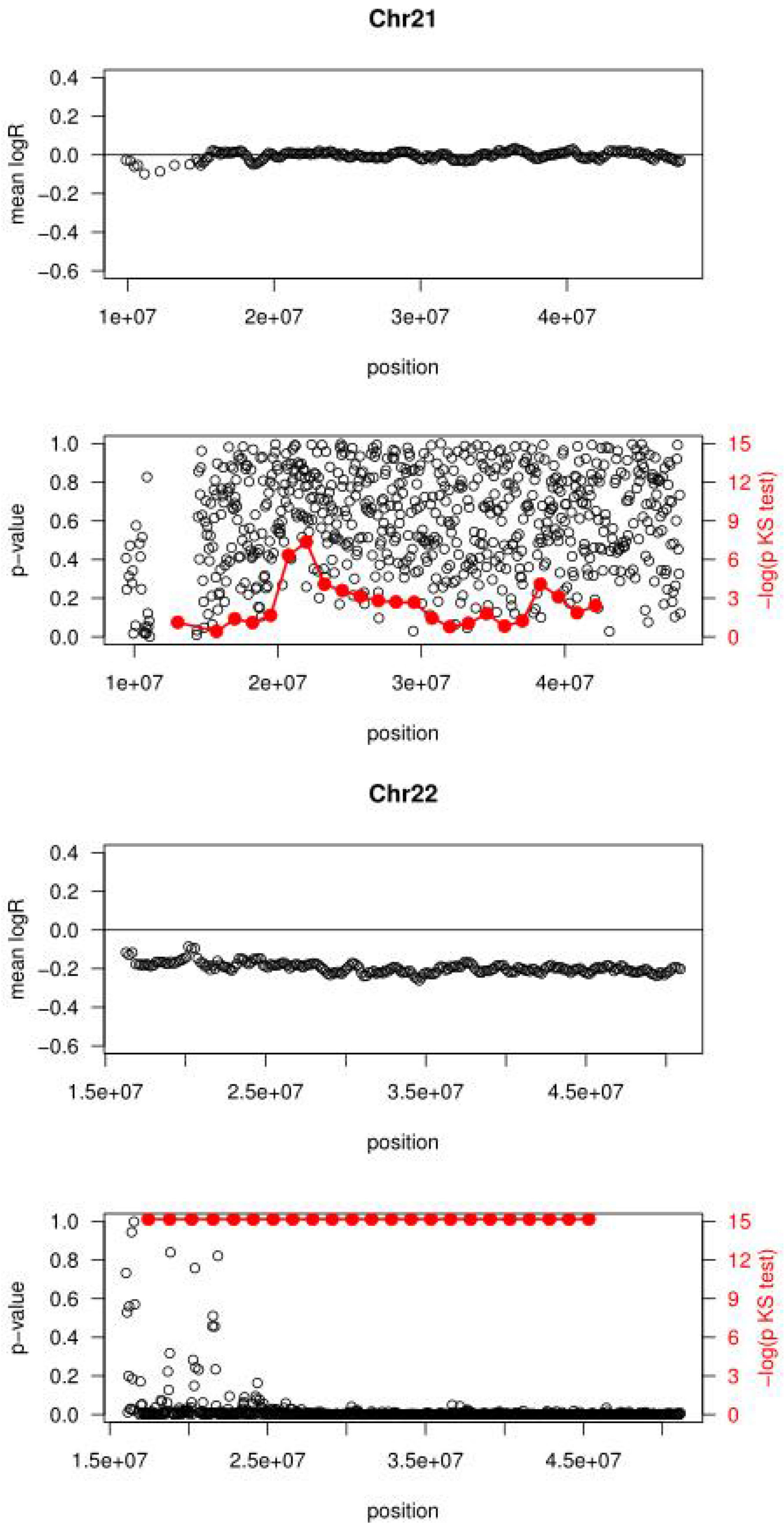

Supplementary Figure 2: Mean copy number profile over the 21 tumor/normal sample pairs. In the upper figures, the mean $\log_2$-ratio over the 21 sample pairs is plotted against chromosome position. In the lower figures, the black dots represent the *p*-values for the one-sample t-test for each 50 kb-bin. The red line represents the negative logarithm of the *p*-value of the Kolmogorov-Smirnov test.

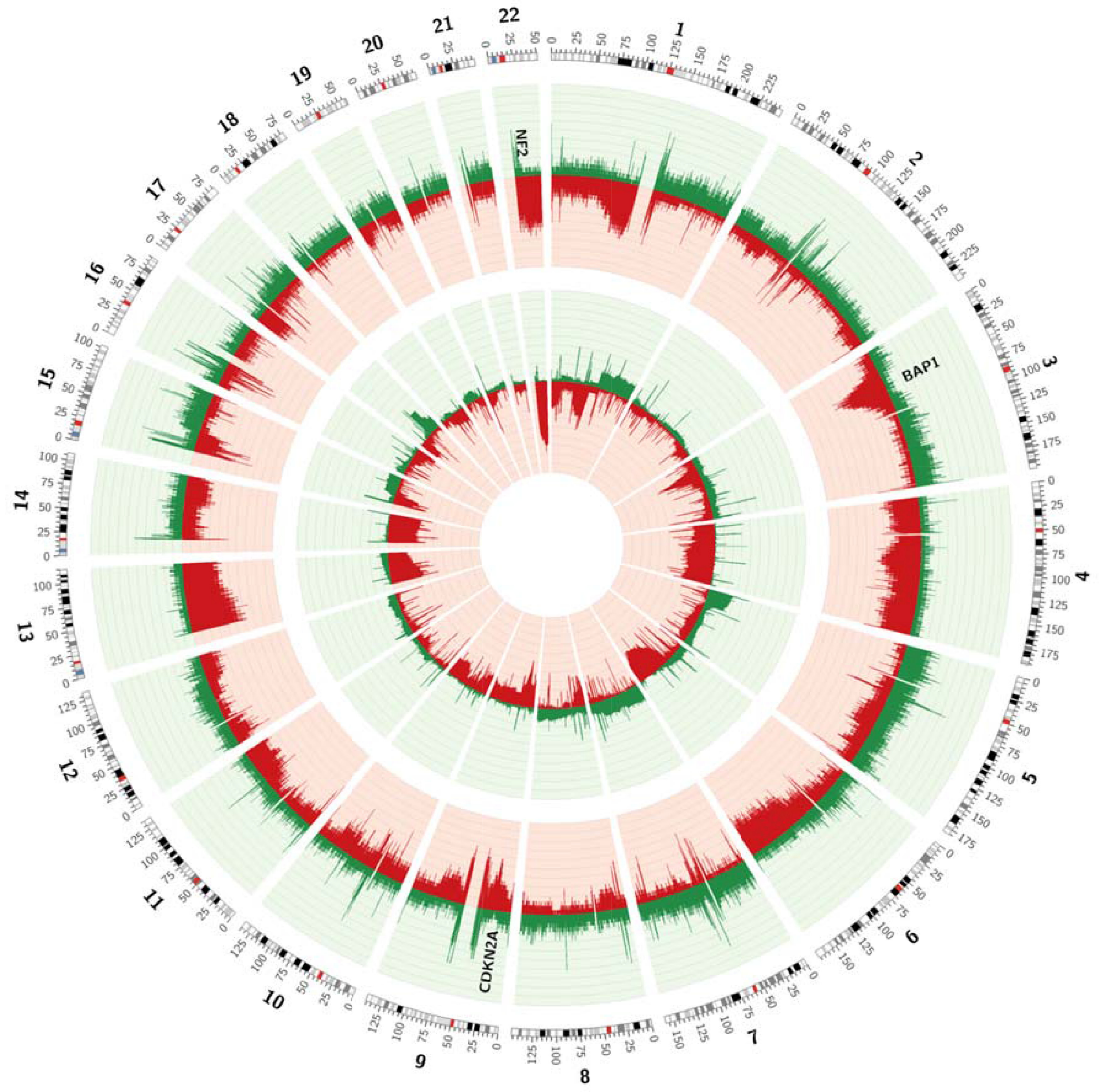

Supplementary Figure 3: Circos plot of the copy number variations observed in low-pass whole genome data of 21 MPMs and array data of 85 MPMs. The outer layer represents the frequency of copy number loss (red) and gain (green) in every 50 kb-bin observed in the low-pass whole genome data. The inner layer represents the frequency of copy number loss (red) and gain (green) for every chromosome position observed in the array data from TCGA.

Supplementary Table 1: Associations between the presence of copy number loss in the 'Cancer census genes' most frequently lost in the TCGA-dataset and clinicopathological parameters

Gene name	*P*-value for association with gender	*P*-value for association with age at diagnosis	*P*-value for association with asbestos exposure	*P*-value for association with histological diagnosis	*P*-value for association with survival
EP300	0.553[a]	0.266	0.749[a]	0.388	1[a]
PDGFB	0.803	0.423	0.749[a]	0.489	0.740[a]
MKL1	0.803	0.423	0.749[a]	0.489	0.740[a]
MYH9	0.803	0.423	0.749[a]	0.489	0.740[a]
APOBEC3B	0.620	0.401	0.529[a]	0.574	1[a]
ZNF278	0.702	0.586	0.826	0.283	0.524[a]
NF2	0.804	0.907	0.525[a]	0.328	0.344[a]
MN1	1	0.542	1	0.291	0.173[a]
CHEK2	0.817	0.749	0.773	0.291	0.322[a]
EWSR1	0.785	0.800	0.928	0.361	0.319[a]
BCR	0.955	0.682	0.677	0.240	0.512[a]
SMARCB1	1	0.553	0.872	0.271	0.340[a]
MAPK1	1	0.633	1	0.428	0.517[a]
CLTCL1	1	1	1	0.623	0.212[a]
SEPT5	1	1	1	0.623	0.212[a]
LZTR1	1	1	1	0.623	0.212[a]
CDKN2A	0.822	0.557	0.958	0.464	**0.010**
SETD2	0.644	0.623	0.141	0.549	0.514[a]
BAP1	1	0.855	0.217	0.945	0.513[a]
NCKIPSD	0.877	0.668	0.134	0.402	0.517[a]
PBRM1	0.835	0.649	0.322	0.945	1[a]

[a]To identify associations, a Pearson's Chi-squared test with Yates' continuity correction was performed. However, when more than 20% of the cells had an expected count below five, a Fisher's Exact test was used.

Supplementary Table 2: Associations between the presence of copy number gain in the 'Cancer census genes' most frequently gained in the TCGA-dataset and clinicopathological parameters

Gene name	*P*-value for association with gender	*P*-value for association with age at diagnosis	*P*-value for association with asbestos exposure	*P*-value for association with histological diagnosis	*P*-value for association with survival
TERT	0.532[a]	0.074	0.736[a]	0.666	1[a]
SDHA	0.528[a]	0.178	0.737[a]	0.778	1[a]
DROSHA	0.514[a]	0.298	0.492[a]	0.909	1[a]
IL7R	0.337[a]	0.469	0.719[a]	1	1[a]
LIFR	0.337[a]	0.469	0.719[a]	1	1[a]
FCGR2B	0.721[a]	0.533[a]	0.678[a]	1[a]	0.675[a]
CDC73	1[a]	1[a]	0.705[a]	0.489[a]	0.188[a]
PTPRC	0.729[a]	1	1[a]	0.721	0.110[a]
MDM4	0.493[a]	1	1[a]	0.764[a]	0.112[a]
ELK4	0.729[a]	1	1[a]	0.721	0.110[a]
SLC45A3	0.729[a]	1	1[a]	0.721	0.110[a]
HLF	1[a]	0.615	0.435[a]	0.336[a]	0.431[a]
MSI2	1[a]	0.557	0.429[a]	0.507[a]	0.408[a]
RNF43	1[a]	0.615	0.435[a]	0.336[a]	0.431[a]
CLTC	1[a]	0.615	0.435[a]	0.336[a]	0.431[a]
PPM1D	1[a]	0.615	0.435[a]	0.336[a]	0.431[a]
BRIP1	1[a]	0.585	0.435[a]	0.338[a]	0.436[a]
CD79B	1[a]	0.615	0.435[a]	0.336[a]	0.431[a]
DDX5	1[a]	0.615	0.435[a]	0.336[a]	0.431[a]
AXIN2	1[a]	0.615	0.435[a]	0.336[a]	0.431[a]
PRKAR1A	1[a]	0.615	0.435[a]	0.336[a]	0.431[a]

[a]To identify associations, a Pearson's Chi-squared test with Yates' continuity correction was performed. However, when more than 20% of the cells had an expected count below five, a Fisher's Exact test was used.

Supplementary Table 3: Associations between the presence of copy number loss in the 'Cancer census genes' most frequently lost in the LP-WGS-dataset and clinicopathological parameters

Gene name	*P*-value for association with gender[a]	*P*-value for association with age at diagnosis[a]	*P*-value for association with histological diagnosis[a]	*P*-value for association with survival[a]	*P*-value for association with neo-adjuvant treatment[a]
EP300	0.500	0.303	1	1	0.362
SETD2	0.533	0.303	1	0.530	1
PBRM1	1	0.303	1	0.530	1
ROS1	0.533	1	1	0.517	0.659
ZNF198	1	0.303	1	0.530	1
RB1	1	0.629	0.521	0.517	0.659
CHEK2	1	0.141	1	1	0.659
TRIM33	0.133	1	1	0.517	1
CACNA1D	1	0.629	1	0.517	1
FLT3	1	0.629	1	0.517	1
FOXO1	1	0.350	0.257	0.263	1
MKL1	1	0.629	1	0.517	1
EPS15	1	0.350	1	0.263	0.387
WHSC1	0.171	1	1	0.263	1
PTPN13	0.171	1	1	0.263	1
RAP1GDS1	0.171	1	1	0.263	1
FBXW7	0.171	1	1	0.228	0.670
FAT1	0.171	1	1	0.228	0.670
NFIB	0.171	0.650	1	0.228	1
MLLT3	0.171	1	0.553	0.263	1
BRCA2	1	1	0.553	0.263	0.670
LHFP	1	0.350	1	0.263	1
LCP1	1	1	1	1	1
MAPK1	1	0.350	0.553	0.263	0.670

[a]To identify associations, a Fisher's Exact test was performed, as for every association more than 20% of the cells had an expected count below five.

Supplementary Table 4: Associations between the presence of copy number gain in the 'Cancer census genes' most frequently gained in the LP-WGS-dataset and clinicopathological parameters

Gene name	*P*-value for association with gender[a]	*P*-value for association with age at diagnosis[a]	*P*-value for association with histological diagnosis[a]	*P*-value for association with survival[a]	*P*-value for association with neo-adjuvant treatment[a]
PMS2	0.533	0.629	0.247	1	1
FCGR2B	0.429	0.629	1	1	0.635
EIF4A2	1	0.629	1	1	0.149
TERT	1	1	0.128	0.530	1
HNRNPA2B1	1	0.582	1	0.530	0.311
EGFR	1	1	0.128	1	1
MET	1	0.582	0.128	1	1
RAD21	1	1	1	0.155	0.635
KLF6	0.429	1	0.549	1	0.635
NAB2	1	0.303	1	1	0.635
MLLT6	0.429	0.303	1	0.530	0.635
CIC	0.429	0.629	0.549	0.155	0.635
FAM131B	1	0.303	1	0.530	1
PLAG1	1	0.582	1	0.530	0.587
CHCHD7	1	0.582	1	1	0.311
RECQL4	0.352	0.303	0.489	1	0.311
NUTM2B	1	1	0.489	1	1
NUTM2A	1	1	1	1	1
ETNK1	1	1	1	0.422	1
DICER1	0.352	1	0.489	0.530	0.311
CD79B	0.352	0.582	0.489	1	1
PRKAR1A	0.352	0.582	0.489	1	0.311
ZNF521	1	1	1	1	1

[a]To identify associations, a Fisher's Exact test was performed, as for every association more than 20% of the cells had an expected count below five.
